# Diversity in emergent cell locomotion from the coupling cytosolic and cortical Marangoni flows with reaction–diffusion dynamics

**DOI:** 10.1371/journal.pcbi.1014216

**Published:** 2026-04-27

**Authors:** Blaž Ivšić, Dorijan Vulić, Igor Weber, Piotr Nowakowski, Ana-Sunčana Smith

**Affiliations:** 1 Division of Physical Chemistry, Ruđer Bošković Institute, Zagreb, Croatia; 2 Centre for Advanced Laser Techniques, Institute of Physics, Zagreb, Croatia; 3 Division of Molecular Biology, Ruđer Bošković Institute, Zagreb, Croatia; 4 Department of Physics, Faculty of Sciences, Friedrich-Alexander-Universität, Erlangen, Bavaria, Germany; 5 Competence Center Engineering of Advanced Materials, Friedrich-Alexander-Universität, Erlangen, Bavaria, Germany; Georg-August-Universitat Gottingen, GERMANY

## Abstract

Cell migration is a fundamental process underlying the survival and function of both unicellular and multicellular organisms. Crawling motility in eukaryotic cells arises from cyclic protrusion and retraction driven by the cytoskeleton, whose organization is regulated by reaction–diffusion (RD) dynamics of Rho GTPases between the cytosol and the cortex. These dynamics generate spatial membrane patterning and establish front–rear polarity through the coupling of biochemical signalling and mechanical feedback. We develop a cross-scale mean-field framework that integrates RD signalling with cytosolic and cortical hydrodynamics to capture the evolution of cell shapes and emergent cellular locomotion. Our model reproduces diverse experimentally observed shape and motility phenotypes with small parameter changes, indicating that these behaviours correspond to self-organized limit cycles. Phase-space analysis reveals that coupling to both cytosolic flow and spatially varying surface tension is essential to recover the full spectrum of motility modes, providing a theoretical foundation for understanding amoeboid migration.

## Author summary

How do free-living amoeboid cells decide where to go when no clear external cues guide them? We developed a computer simulation that mimics how simple cells move freely across a flat surface. The model connects simple chemical reactions within the cell cortex with the flow on the cell surface and of its inner fluid. In this way, the shape of cell changes and locomotion is induced. Our simulations show that this internal coordination alone can produce motion patterns seen in real cells—steady gliding, shifting directions, circling, or turning intermittently. By tuning key coupling parameters, we found how small internal changes can trigger very different behaviours. The study hence reveals how, based on simple physical principles, cells can self-organise and explore their surroundings.

## Introduction

Cell migration is a fundamental biological process essential for the survival of both unicellular and multicellular organisms, spanning from flagellated bacteria navigating liquid environments to motile fibroblasts orchestrating wound closure in animals. Crawling motility in eukaryotic cells relies on cyclic protrusion and retraction driven by the cytoskeleton, a composite network of actin filaments, microtubules, and intermediate filaments [1,2]. Coordination among these structural components is mediated by a complex signalling network, prominently the Rho family of GTPases, which act as molecular switches regulating cytoskeletal dynamics [3]. In their active, GTP-bound state these proteins transmit signals to downstream effectors that locally modulate filament assembly and contractility [4]. Even in the absence of external stimuli, Rho GTPases can self-organize through reaction–diffusion (RD) dynamics operating between the cytosol and the cell cortex [5–7]. Such dynamics give rise to spatial membrane patterning with localized zones of high activator concentration that promote actin polymerization and define cell polarity. The interplay between cortical and cytosolic flows, together with RD feedback, establishes a tightly coupled system in which biochemical signalling and mechanical deformation co-regulate cell shape, polarity, and motility in a continuously self-organizing manner.

Numerous approaches have been proposed to model such a complex biological task [8–11]. A variety of approximations and computational techniques can be employed to model each of the aforementioned processes with varying levels of biological complexity and precision. Main difference among these approaches lies in the way forces are incorporated with the RD system and in the way interface and the position of the cell is tracked. Before outlining the methods available for tracking shape and translating the cell body, we briefly introduce the approach to model signalling dynamics.

Since Turing’s pioneering work [12], which demonstrated that two interacting and diffusing substances can form stable spatial patterns in their concentration distribution, RD systems have become an indispensable approach for modelling numerous biological processes. Moreover, RD systems extend Turing’s original idea by revealing not only stationary but also time-dependent dynamics, which can explain cyclic biological processes [13]. It has been shown repeatedly that RD systems, based on known interactions of the Rho family of GTPases and related proteins, can explain stable polarization [14,15] as well as oscillatory dynamics [6,16,17].

The aforementioned dynamics directly influence how the cytoskeleton is organized. To model the motility process, one thus needs to couple RD system to a component which tracks and translates the cell. The simplest way of approaching this task is to track only the cell contour on a two-dimensional domain. Such model was proposed by Nielson et al. [18]. The authors combine Parametrized Finite Element Method (PFEM), used to track and evolve the interface, with a simple RD system which governs the normal velocity of the cell membrane. The main drawback of such an approach is the fact that tangential evolution of the nodes, which form the contour of cell, is controlled by Moving Mesh Partial Differential Equations (MMPDEs), which do not necessarily align with realistic biological mechanics.

Apart from tangential evolution, another shortcoming of this approach is the lack of cytoplasmic dynamics inside the cell. To address this, two upgraded models similar to PFEM were proposed [19,20]. Both approaches employ explicit bulk and surface tracking by evolving the positions of nodes that define the interior, exterior, and cortex of the cell. The main advantage of these so-called Arbitrary Lagrangian–Eulerian (ALE) approaches is their reduced computational cost compared to other high-resolution models, although this comes at the expense of a mathematically complex formulation of the MMPDEs governing node translation. However, ALE-based models struggle to fully capture membrane tension and intracellular forces that influence the bulk nodes. Instead, these models resemble mechanical spring-force approximations.

Another type of models that usually incorporate the RD dynamics with the changes in cell shape better than the previously mentioned ones are Cellular Potts Models (CPM). In these models, Monte Carlo algorithm is used to evolve the cell interface. Maree et al. [21] implemented CPM with a highly complex signalling network, incorporating multiple protein species and their interactions. This highlights the primary strength of the CPM approach: it can be easily coupled with intricate biochemical networks to model a wide range of biophysical processes involved in cell motility, while maintaining a relatively low computational cost. However, a key limitation of CPM is that the shape evolution dynamics are inherently stochastic and do not necessarily reflect the physical nature of the forces driving cell motility.

To address the stochastic nature of shape changes in CPM, one can employ a Phase Field (PF) model. This class of models naturally integrates free energy formulations into their evolution equations, improving the physical description of the system, and has been widely used for simulating cell motility [22–26]. PF models fall into the category of diffuse interface models, meaning that the cell is represented by a smooth phase-field function that transitions gradually across the simulation domain. On the downside, the diffuse interface imposes a lower limit on grid spacing and resolution to ensure accurate representation of the phase-field transition from the cell interior to its exterior.

The drawback of all of the aforementioned models lies in the fact that they do not account for the flow inside the cytoplasm which can affect the RD system in moving cells. This can be mitigated by the use of an approach similar to PF called the Level-Set (LS) method. Originally developed for simulating phase flow problems [27], this technique has been effectively applied to modelling of cell migration. Within LS formalism, the cell boundary is naturally embedded within a continuous field. Notably, this formulation assumes that the cortex is a one-dimensional curve of zero thickness. Several studies have successfully employed LS methods for cell motility simulations [28–30], each incorporating different techniques to model signalling dynamics. The main advantage of the LS approach, particularly compared to the previously mentioned methods, is its ability to simulate highly irregular cell shapes and their dynamic deformations. However, a key drawback is the gradual degradation of the LS function over time.

The explicit interface tracking of the LS method can itself be challenging, especially when trying to incorporate RD systems that have defined surface protein concentration and their diffusion. An alternative approach [31], which combines elements of both LS and PF methods, is to define the LS as smooth function across the interface (such as a hyperbolic tangent), rather than a step or a signed distance change. Although the interface position is still determined by a predefined contour level, it is now diffuse rather than sharp.

In this paper, we propose a minimal model of cellular locomotion that combines different aspects of the aforementioned approaches. To account for cytosolic flow, cell shape, and RD dynamics [32], we link the LS method with the Navier-Stokes equation to track cell position and account for cytosolic and cortex flows, and couple these components to the canonical RD system. This allows for simulation of non-stimulated cell motility as well as chemotactic locomotion (with minimal updates to the model).

## Model

**Fig 1:**
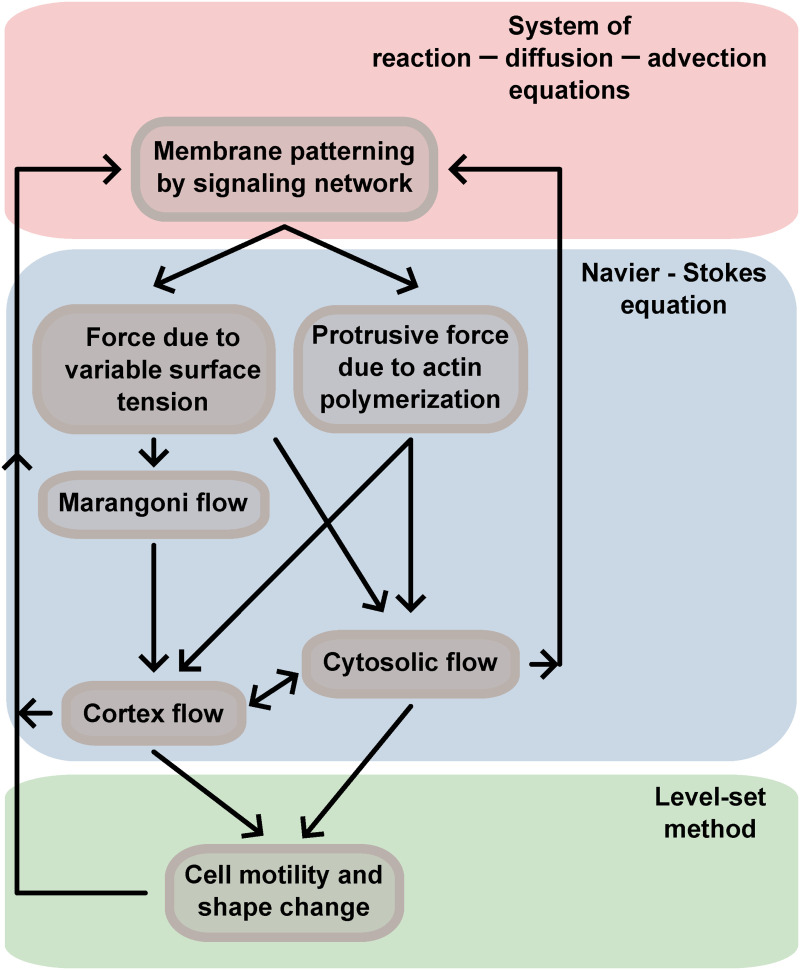
Diagram of the components of the model. To fully capture complex biological process of cell motility one needs to couple membrane patterning by reaction–diffusion–advection network to forces which induce cytosolic and membrane flows as well as facilitate shape change and motility in general. The coupling loop is closed by allowing the generated flows and geometry of the cell to influence reaction–diffusion–advection system.

We propose a model of cell locomotion that combines LS formalism with fluid flow and reaction–diffusion–advection dynamics. The components of the model and the relations between them are shown schematically in Fig 1.

### Level-set formalism

Motivated by two-phase-flow applications, we adopt a LS approach, specifically a diffuse-interface variant [33]. This method allows for separation of different regions with a scalar field ϕ, referred to as LS function. The evolution of this function is governed by the conservative continuity equation [27,34]


$$\frac{\partial \phi }{\partial t}+\nabla \cdot (v\phi )=0,$$ (1)

which transports ϕ under the prescribed velocity field v, and we use *t* to denote time. For incompressible fluids, as considered herein, we note that this approach is equivalen*t* to the classical LS method.

Given the freedom to specify the shape of the LS function, we adopt a diffuse-interface variant, common in phase-field (PF) models [35]. We model the shape by demanding ϕ=1 inside and ϕ=0 outside the cell, with a continuous smooth function connecting these two regions in the shape of hyperbolic tangent [31,35]


$$\phi \left(r,\theta \right)=\frac{1}{2}\left[1-\tanh\left(\frac{r-r_{0}\left(\theta \right)}{\epsilon /3}\right)\right],$$ (2)

where, for the sake of clarity, we have used polar coordinates (r,θ), and assumed that the location of the interface can be described by an angle-dependent distance as $r_{0}(\theta )$. The parameter ϵ controls the width of the crossover.

Employing a hyperbolic-tangent profile (2) creates an interface region of thickness ϵ which allows us to model membrane processes within our two-dimensional framework. To this end, we define [36]


$$\delta_{\epsilon }\left(r\right)=\left|\nabla \phi \right|$$ (3)

as a numerical approximation of the Dirac delta function supported on the interface line ϕ=1/2, where the vector r denotes a point on the plane. Since the integral of $\delta_{\epsilon }$ in the direction perpendicular to the interface is 1, this function can be used to localise biochemical processes in the cell cortex. Furthermore, in the diffuse-interface framework geometric quantities, such as normal vector or interface curvature, can be derived from derivatives of ϕ [33] (see Section A in S1 Appendix for details).

A well-known drawback of this approach is a slow degradation of the level set function profile by advection (Eq (1)) [37]. We restore the desired profile by repeatedly performing reinitialisation, *i.e.*, by evolving ϕ in a pseudo-time τ between physical time steps, using the conservative scheme, which was developed for the application in conjunction with the continuity equation (1) by Parameswaran and Mandal [38].

Finally, we note that the widening on the interface in the LS approach requires us to treat various surface quantities as volume ones, including surface density of activator which is measured in 1/µm^3^ units, see Section B in S1 Appendix for the detailed discussion of this problem.

### Fluid dynamics

For the evolution of the velocity field v we use the Navier–Stokes equation [39]


$$\rho \frac{\partial v}{\partial t}=-\nabla p+\nu \nabla^{2}v+f,$$ (4)

where ρ is the density of the fluid, ν is the dynamic viscosity, *p* is the pressure, and f denotes external or internal forces acting on the fluid. In the equation above, we neglect the non-linear term as we are interested in low-Raynolds-number flows, which also renders the left hand side of the equation negligible, and we assume that the liquid is incompressible (∇·v=0) which determines the pressure *p*.

We assume that the force density acting on the fluid can be decomposed as [40–44]


$$f=\left[\sigma \kappa \hat{n}+\nabla \sigma -\hat{n}\left(\hat{n}\cdot \nabla \sigma \right)+k_{C}C_{a}\hat{n}-\alpha \left(A-A_{0}\right)\hat{n}\right]\delta_{\epsilon }\left(r\right)-\beta v.$$ (5)

where σ denotes surface tension, $\hat{n}=-\nabla \phi /\left|\nabla \phi \right|$ is a unit vector normal to the interface, $\kappa =\nabla \cdot \hat{n}$ is the local curvature of the interface, $C_{a}$ is the concentration of activator species (see below), and *A* denotes the area of the cell. In the above equation $k_{C}$, α, *A*_0_, and β denote parameters of the model which we discuss below, and the factor $\delta_{\epsilon }\left(r\right)$ ensures that forces described by all terms but last only act on the interface.

In Eq (5) the first term ($\sigma \kappa \hat{n}$) describes the normal force resulting from the surface tension σ. We assume a linear dependence


$$\sigma =\sigma_{0}+k_{\sigma }C_{a},$$ (6)

such that the surface tension is modified from its base value $\sigma_{0}$ by a term proportional to the concentration of the activator $C_{a}$ with the coefficient $k_{\sigma }$. The space-dependent surface tension induces the Marangoni surface flow [41,45,46] (from regions of lower to higher surface tension) described by the second and third terms ($\nabla \sigma -\hat{n}\left(\hat{n}\cdot \nabla \sigma \right)$) in Eq (5).

The idea of spatially dependent cortical tension generated by the network of actin filaments has been well established in the literature [47–51], and recently considered in the context of cell motility However, to the best of our knowledge, its effect on cortical flow has not been studied numerically in the context of cell motility, except for a recent study of a cell moving in a narrow channel due to external anisotropy coupling to the myosin concentration [52].

The fourth term in Eq (5) ($k_{C}C_{a}\hat{n}$) represents the protrusive force generated by actin polymerisation at the leading edge [42,53]. We assume that this force is proportional to the concentration of activator $C_{a}$ with coefficient $k_{C}$. The fifth term ($-\alpha \left(A-A_{0}\right)\hat{n}$) enforces the near-constancy of the projected cell area $A\approx A_{0}$, as observed experimentally [43]. Finally, the linear drag term −βv models the dissipation and ensures that the flow relaxes when external forcing stops [44].

Before proceeding, we briefly justify the specific structure of the force density postulated in Eq (5). First, the Marangoni flow can strongly bias the otherwise diffusive transport of membrane-bound proteins [54,55]. Second, letting both surface tension and protrusive force depend on local activator concentration directly couples cortical mechanics to the biochemical polarity cue: high activator activity marks the protruding edge of the cell [56,57]. Because the leading and trailing edges exhibit different membrane tensions [41,57], we represent relative changes in σ with the activator-dependent law (6) rather than adding further geometric factors. Finally, the actin-generated protrusive force is approximated to act normally on the cortex, consistent with the mean orientation of the filament polymerisation against the membrane [42]. Eqs (4)–(6) thus provide a minimal, yet mechanistically grounded, route by which the distribution of activator modulates the cell shape as well as intracellular and surface flows.

### Reaction–diffusion–advection system

The objective of the reaction–diffusion–advection (RDA) model is to capture the dynamics of activator molecules whose concentration is coupled to the force profiles in the cell cortex and to the substrate molecules from which the activator is produced [12]. These coupled species constitute the core of a minimal Turing-type activator–substrate model capable of producing stationary polarity patterns [16,58]. To extend the available solution space to include time-dependent (Hopf-type) and wave-pinning solutions, we need to introduce at least one additional protein species. Moreover, altogether at least four interacting protein species are required to reproduce all of the above solution types [59].

In the current work, we used *Dictyostelium discoideum* as an example system for which RD equations have been shown to reproduce experimentally observable activator dynamics in stationary cells [6]. We opt for a reduced version of the original model consisting of cytosolic substrate and inhibitor, a membrane-bound activator, and a complex between the activator and its inhibitor. Such interaction network can be easily related to the Rho GTPase switching cycle [7,60–62]. For example, the role of the activator can be attributed to the active form of a GTPase, while its cytosolic substrate can be related to its inactive form bound to RhoGDI. Biologically, the signalling cycle ends with the deactivation of the GTPase mediated by an inhibitor protein, GAP, and in our model this interaction is modelled by formation and dissociation of the activator–inhibitor complex.

Based on this rationale, we define four protein species. Two are cytoplasmic, *i.e.*, restricted to the bulk region, and two are constrained to the cell cortex. The membrane species that plays a role of the activator that couples to the forces is denoted by the index “a”. The bulk species, that acts as the substrate for this activator, is denoted by index “s”. These two species comprise a minimal Turing-type model. To expand the solution space with wave-pinning-type solutions, we need to add a delayed inhibition. This is achieved by introducing a second membrane species that also catalytically promotes the formation of the activator, which we denote by the index “c”, representing a complex formed from the activator and another protein. The partner required to form these complexes is an inhibitor species restricted to the bulk, which we denote by the index “in”.

**Fig 2:**
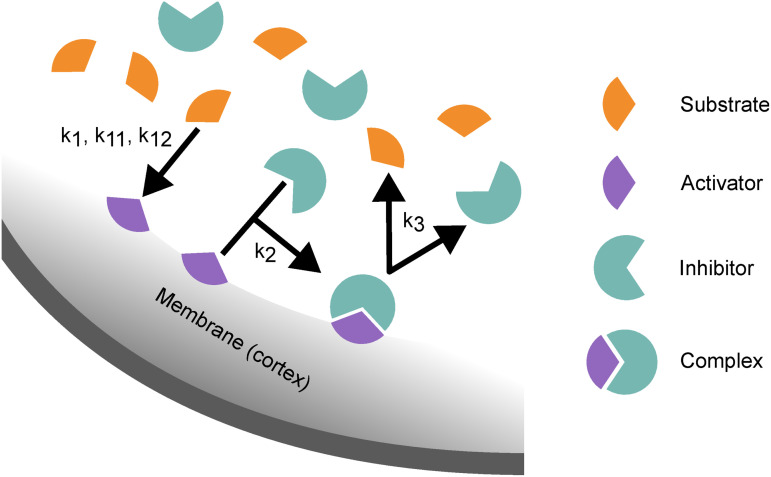
Schematic representation of the reactions of the model. Arrows indicate the direction of the reaction with governing constants for that reaction annotated beside them. Cytosolic substrate binds to the membrane becoming the activator. Membrane bound activator can form a complex with the cytosolic inhibitor. The cycle ends when the complex dissociates, releasing substrate and inhibitor back into the cytosol.

We consider three reactions of the species: First, a cortex-bound activator “a” is produced autocatalytically from the bulk substrate “s”. Second, the activator “a” together with the bulk inhibitor “in” can form a membrane-bound complex “c”. Finally, the complexes can dissociate back into two bulk species. These reactions are presented schematically in Fig 2. Each species diffuses freely, either two-dimensionally in the bulk cytoplasm or, effectively, one-dimensionally along the membrane cortex, and the reactions are thus restricted to the overlap between the diffuse-interface region and the bulk. For each of the species we introduce a bulk concentration field $C_{i}\left(r,t\right)$, where i=a,c,s,in denotes the type of species. We use the following mass-conserving reaction terms for each of the species in the RDA equations:


$$R_{s}=k_{3}C_{c}-C_{s}\left(1-\frac{C_{a}}{C_{a}^{\text{MAX}}}\right)\left(k_{1}+k_{11}C_{a}+k_{12}C_{c}\right),$$ (7a)

$$R_{in}=k_{3}C_{c}-k_{2}C_{a}C_{in},$$ (7b)

$$R_{a}=-k_{2}C_{a}C_{in}+C_{s}\left(1-\frac{C_{a}}{C_{a}^{\text{MAX}}}\right)\left(k_{1}+k_{11}C_{a}+k_{12}C_{c}\right),$$ (7c)

$$R_{c}=k_{2}C_{a}C_{in}-k_{3}C_{c},$$ (7d)

where we have introduced several reaction rates: *k*_1_ is the basal rate at which the cytosolic substrate “s” binds and activates on the membrane, *k*_11_ and *k*_12_ quantify the co-operative autocatalysis mediated, respectively, by the membrane activator “a” and the transient complex “c”. The positive feedback provided by *k*_11_ supplies the classical activator self-activation mechanism of Turing-type systems [12,58]. The additional delayed feedback through *k*_12_ (activator “a”, that is momentarily inactive within the complex “c”, still promotes further activator “a” recruitment) has been shown to broaden the parameter range that supports pattern formation in mass-conserved models [15,63]. Complex formation and dissociation are governed by *k*_2_ and *k*_3_, respectively. Finally, the factor $\left(1-C_{a}/C_{a}^{\text{MAX}}\right)$ prevents the activator “a” from exceeding a limit of steric saturation on the membrane [63,64].

The postulated reaction rates allow us to write the RDA equations for all species:


$$\frac{\partial C_{i}}{\partial t}+\nabla \cdot \left(vC_{i}\right)=\nabla \cdot \left(D_{i}\nabla C_{i}\right)-\nabla \cdot \left(\frac{D_{i}}{k_{B}T}F_{i}C_{i}\right)+R_{i},$$ (8)

such that each concentration field $C_{i}$ is advected with velocity v, diffuses with constant $D_{i}$, interacts with external force $F_{i}$, and is subject to reactions given by $R_{i}$ in Eq (7). In the above equation, the index i=a,c,s,in denotes the type of species, $k_{B}$ is the Boltzmann constant, and *T* is the temperature.

The role of the introduced forces $F_{i}$ is to restrict surface and bulk species to the desired domains. To this end, we use $F_{i}=-\nabla U_{i}$, and assume


$$U_{s}=U_{in}=-k_{B}T\ln(\phi \left(r\right)),$$ (9a)

$$U_{a}=U_{c}=-k_{B}T\ln\left(\delta_{\epsilon }\left(r\right)\frac{2\epsilon }{3}\right),$$ (9b)

such that in the equilibrium, without reactions, the probability distribution becomes proportional to ϕ(r) or $\delta_{\epsilon }\left(r\right)$ for bulk or surface species, respectively. We have chosen the confining-force method of restricting species over the pointwise Dirichlet constraints because this method strictly preserves mass conservation of each of the species and remains compatible with the diffuse-interface formulation of LS function.

Finally, we note that reactions between cytosolic and membrane species are possible only in the finite overlap zone of interface and bulk confining potentials. The reaction rates in our model can be related to those in models with a one-dimensional interface [6] by integrating the local kinetics across the overlap region, a procedure analogous to the surface–volume coupling used in diffuse-interface models of phase flows with soluble surfactants [65].

## Methods

To analyse our model, we have solved the relevant equations numerically using well established methods of computational physics [31,66–68]. We considered a square region with periodic boundary conditions in which we discretised all relevant fields of our model. Then, all spatial derivatives were replaced with finite differences and we advanced system in time using forward-Euler time integration procedure. The details of this procedure are presented in Section A in S1 Appendix.

The calculations were performed using a specially written computer programme in ISO C99 [69]. The parameters were chosen to follow the experimental measurements and the numerical works studying *Dictyostelium discoideum* [6,70–81]. In the simulations we varied coupling constants $k_{C}$ and $k_{\sigma }$, as well as diffusion constants $D_{a}$ and $D_{c}$. The complete list of the values of the parameters used in the simulations is presented in Tables A–D in S1 Appendix.

Special attention was paid to the initial configuration of the concentration fields for all species. To this end, we performed preliminary simulations in which the circular shape of the cell was fixed by blocking the evolution of the LS and velocity fields. As we observed, in this case, the RD dynamics can generate three different patterns of proteins: oscillations, rotations, and stable polarisation [82–85]. To speed up our simulations, we have used the oscillatory and rotational patterns established in the preliminary simulations as initial configurations. The details of the preliminary simulations are discussed in Section C in S1 Appendix.

The analysis of the results was performed using commercially available programmes [86–88]. By finding the centre of mass for each snapshot, we determined the *velocity* of the cell and plotted its *trajectory*. Cell *elongation* was calculated as $E=\log_{2}\left(a/b\right)$, where *a* and *b* denote the axes of an ellipse fitted to the cell shape [89,90]. The profile of the activator concentration $C_{a}$ was quantified by the *polarity vector*. In the case of a single activator patch in the cortex, this vector points from the centre of the cell toward the patch. Finally, to study the *flow field*, we transformed the velocity v to the centre-of-mass reference frame and, using the LS function, split the flow into surface and cytosolic parts. The details of our analysis are reported in Section A in S1 Appendix.

## Results: Properties of the solutions

### Flows induced by protrusive coupling and varying surface tension

We start analysis of the solutions of our model by studying the effect of the activator on the flow. In Eq (5) there are two terms dependent on $C_{a}$: the protrusive force with coupling $k_{C}$, and the surface tension forces via concentration-dependent σ (see Eq (6)) with a coupling constant $k_{\sigma }$.

#### Protrusive coupling flow.

**Fig 3:**
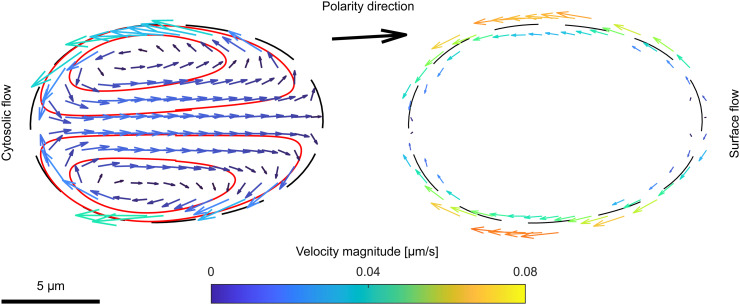
Flow induced by protrusive coupling. Cytosolic and cortex flows, in the centre-of-mass reference frame of the cell, during movement under the influence of only protrusive coupling for $k_{C}=0.04\times 100\;pN\;\mu m$ and $k_{\sigma }=0$. The black arrow marks the polarity direction (and, consequently, the direction of motion as well as the position of the patch of activator). Red lines represent streamlines inside the cytosol. The colour scale for both plots is shown in the middle. Left panel shows cytosolic flow and right panel presents cortex flow induced by protrusive coupling.

We first study the effect of protrusion forces that are modelling the effects of actin filaments in real cell. To this end, we have initiated a series of simulations with $k_{C}>0$ and $k_{\sigma }=0$ (*i.e.*, with constant surface tension $\sigma =\sigma_{0}$). We started from a circular shape of the cell and used an oscillatory initial protein concentration profiles. Under these conditions, when $k_{C}$ is large enough, the simulated cell elongates into elliptical shape and moves in a fixed direction defined by the location of the activator patch on the cortex. In Fig 3 we present a plot of a typical flow observed in this case.

As shown in the left panel of Fig 3, inside the cytosol the flow forms two vortices—the fluid is carried from the front (right side of the plot) to the rear of the cell along the top and bottom part of the cortex, the two flows meet at the back and turn, transporting fluid from the rear to the front along the long axis of cell. Furthermore, inspection of the streamlines reveals that particles trapped within the vortices seldom leave the closed loops along which they circulate. This observation is in a full agreement with both experimental and previously reported numerical studies [91,92].

In the right panel of Fig 3 we show the cortex flow. It is strongly affected by the movement of the cell: in the reference frame of the cell, the fluid streams along the cortex, inducing stress as it is dragged along the interface. The resulting cortex flow runs from the front to the rear of the cell along the perimeter with a varying magnitude—low in front and back of the cell and high in the middle regions.

The induced cortex flow carries cortical protein species along the perimeter from the position of the patch of activator. As a result, the diffusive transport of proteins from the front to the rear is enhanced. At the same time, the cytosolic flow inside transports substrate species from rear directly to the front along the long axis of the cell, facilitating the polarization and maintaining the patch. This mechanism facilitates static polarization and subsequent persistent motility [93–95].

#### Surface tension coupling flow.

Motile cells are typically polarized, exhibiting a front-rear asymmetry in cortex mechanics (often higher cortical tension at the rear for cell-body retraction) while advancing via actin-based protrusions such as filopodia or pseudopodia at the front [96]. To capture this behaviour, our model includes a direct coupling between activator concentration $C_{a}$ and cortex tension σ regulated by the parameter $k_{\sigma }$ (see Eq (6)), which may be both positive or negative, leading to systems with either higher or lower frontal cortex tension, respectively.

The flow arising from variable surface tension, *i.e.*, the Marangoni flow, is directed from regions of lower surface tension toward regions of higher surface tension. Consequently, for positive coupling, the flow is directed toward the maximum activator concentration, while for negative coupling, it is directed in the opposite direction. We refer to these two cases as a *constrictive* flow (for $k_{\sigma }>0$) and *dispersive* flow (for $k_{\sigma }<0$), respectively.

**Fig 4:**
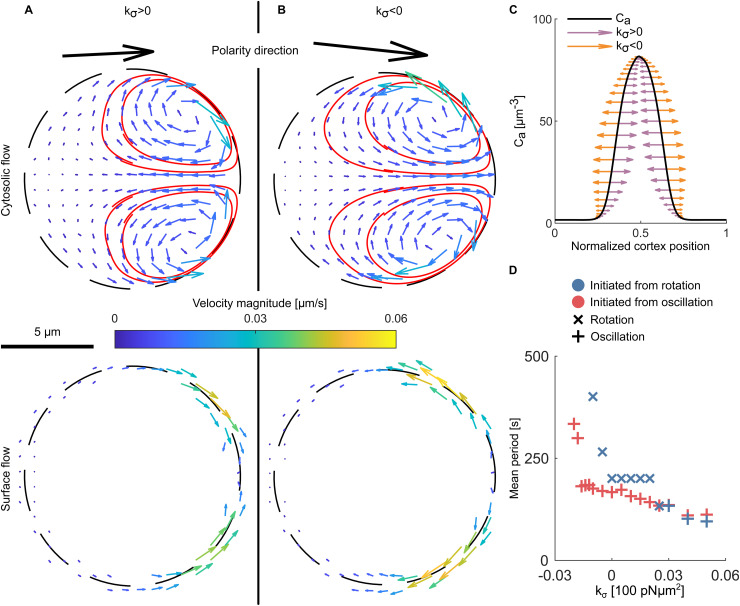
Constrictive and dispersive flow. The flow induced in the cell without protrusion force ($k_{C}=0$) (A) for a positive surface tension coupling $k_{\sigma }=0.03\times 100\;pN\;\mu m^{2}$, and (B) for a negative coupling $k_{\sigma }=-0.03\times 100\;pN\;\mu m^{2}$. In both panels the patch of activator is located on the right side of cell and the snapshot has been taken in the initial phase of the simulation, where the effect of Marangoni flow is the best visible. In both panels the flow field is presented with arrows and streamlines are denoted with red lines. The magnitude of velocity is also shown with the colour code as presented on the scale bar in the middle of panels. Top and bottom graphs show the cytosolic and the cortex flows, respectively. **(C)** The effect of Marangoni flow on the profile of activator concentration along the cortex. Purple arrows refer to positive surface tension coupling while orange ones represent negative surface tension coupling. **(D)** Mean period of the rotation or oscillation RD dynamics as a function of surface tension coupling. Colour marks the type of initial concentration profile for each simulation. Markers represent the observed limit cycle RD pattern obtained after 1300 s of simulation time. Mean period was averaged over 900˗1300 of simulation time. Higher values of surface tension coupling speed up the RD dynamics and favour oscillation pattern. After the threshold of $0.03\times 100\;pN\;\mu m^{2}$ is passed, limit cycle solution of simulations initiated with rotation pattern becomes oscillation. We note that the snapshots present the flow in early stages of simulation, where the flow have not managed to deform the shape of cell significantly.

To analyse in detail the effect of this coupling, we initiated two simulations starting from a circular cell with oscillatory initial concentration profiles without protrusion forces ($k_{C}=0$) and with positive and negative $k_{\sigma }$, respectively. The typical flows induced inside the cytosol and along the cell cortex are shown in Fig 4. As these cells do not necessarily form static polarization patterns, to illustrate the properties of flow profiles in the best possible way, the presented snapshots are from initial stages of simulations, before the flow manages to deform the shape of the cortex and other mechanisms become relevant.

In Fig 4A, we present the constrictive flow induced in the cytosol and in the cortex for positive coupling $k_{\sigma }>0$. The Marangoni flow carries fluid along the cortex toward the activator patch at the front of the cell (right side of cells in Fig 4). The fluid cycles back to the rear along the *x*-axis, forming two vortices. The flow is the strongest in the cortex near the top and bottom boundary of the patch of activator, where the gradient of surface tension is the strongest. In contrast with the case of protrusive flow (see Fig 3), in the present case, the streamlines are concentrated at the front of the cell, indicating that transport in the rear part is negligible. For the dispersive case of negative coupling $k_{\sigma }<0$, as shown in Fig 4B, the flow is naturally reversed: fluid moves toward the front along the *x*-axis and returns to the rear along the cortex. However, the streamlines reveal that the overall characteristics remain the same with two vortices located at the front of the cell. The consequence of localised constrictive or dispersive flow is that the rear of the cell becomes largely segregated from the dynamics of the system as the transported proteins can bypass the rear part of the cell in their cycling.

Despite their similarity, these two cases differ in the direction of the flow which makes the dynamics of the system inherently different. In Fig 4C, we present a typical snapshot of the activator concentration profile along the cortex, with arrows indicating the magnitude and direction of the force generated by the Marangoni effect for the constrictive (purple) and dispersive (orange) cases. The force is largest at midpoints of the slopes on either side of the peak, where the gradient of $C_{a}$ is the largest. In the dispersive case, it acts to bulge out and widen the peak, whereas in the constrictive case to compress and sharpen it.

In the case of dispersive flow (negative $k_{\sigma }$), the Marangoni effect supports the diffusive transport of activator on the cortex. Indeed, the flow profiles in Fig 4A and Fig 3 are similar and, if the coupling $k_{\sigma }$ is negative enough, we observe a similar behaviour of cells with a stable patch of activator in front and a slow straight movement (though with a slightly different shape of cortex caused by different flow patterns).

In contrast, constrictive flow (positive $k_{\sigma }$) counteracts diffusion, effectively squeezing the patch, while the cytosolic flow carries bulk species away from the front and delivers them toward the sides of the cell. This makes the pattern of activator on cortex unstable and promotes oscillatory dynamics. To explain this effect it is useful to consider the oscillation as two wave packets travelling in opposite directions along the cortex. When these packets meet, they form a large peak; when separated, a trough forms between them. As shown in Fig 4C, the constrictive force pushes the packets toward each other and, thus, increases their propagation speed. Consequently, constrictive flow accelerates the oscillation dynamics, reducing the oscillation period as the coupling strength increases (Fig 4D). In contrast, dispersive flow slows oscillations at small negative $k_{\sigma }$ and, upon further increase of the negative coupling, eventually, abolishes them entirely once a certain threshold of $k_{\sigma }$ is reached.

Rotation dynamics respond differently to the two flow types. Under constrictive flow, rotation transitions to oscillation once a positive coupling threshold is exceeded (around $0.03\times 100\;pN\;\mu m^{2}$ in Fig 4D). This arises from the inherent asymmetry of the rotating peak: its leading edge (facing the direction of motion) is steeper than its trailing edge. This asymmetry generates a left–right force imbalance, with the constrictive flow preferentially accelerating waves travelling opposite to the motion of the peak. This rebalances the strengths of clockwise and counter-clockwise modes, ultimately shifting the pattern from rotation to oscillation. In contrast, strong dispersive flow affects rotation in the same way as oscillation, driving the system into static polarisation once a negative coupling threshold is surpassed.

### Motility modes and cell shape phenotypes

**Fig 5:**
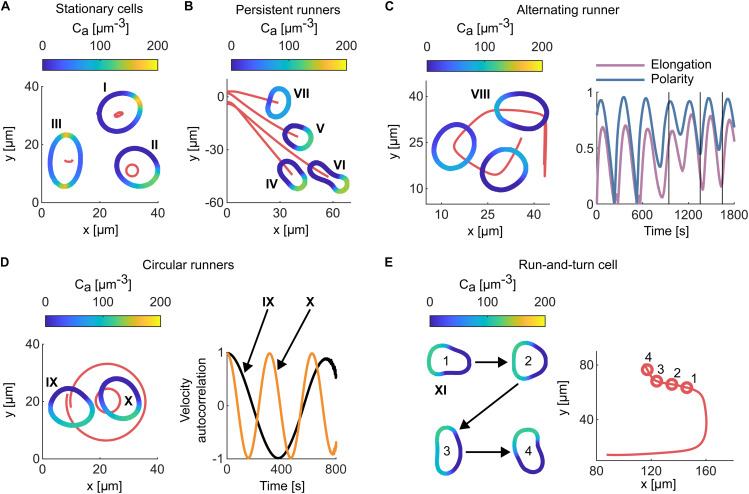
Characteristic motility modes and shape phenotypes. Colour codes denote the concentration of activator in the cortex (as shown by scale bars). Trajectory of the cell geometric centre is plotted with red colour for (A) stationary cells, (B) persistent runner cells, (C) alternating runner cells and the dependence of their time evolution of elongation and polarity. Vertical black lines denote moments in which the plotted snapshots were taken. **(D)** Circular runner cell’s trajectories and the associated velocity autocorrelation functions. **(E)** Run-and-turn cell trajectory is plotted separately on the right side and four circles mark the points in which snapshots plotted on the left side were taken. Videos showing the time evolution of presented cells, are provided in the Supporting information Section. The parameters for these simulations are given in Table D in S1 Appendix.

In our simulations, by varying the strength of the couplings, number of proteins, and initial activator profiles, we have managed to generate a wide range of distinct behaviours of the cell. In order to describe them, it is useful to introduce a categorization into separate motility classes. In our analysis, we have used five distinct classes as presented in Fig 5.

#### Stationary cells.

We start from the stationary cells, *i.e.*, those that are practically not moving. Formally, they are defined as those cells for which the geometrical centre remains inside the cell for the whole duration of the simulation. Of course, the lack of movement does not imply that the cell reaches a stationary state of the RD system. Typically, the concentration of activator on the cortex experience rotational (patch is moving around the cortex) or oscillatory (patch is disappearing and appearing on the cortex) dynamics which is accompanied with changes of the shape of cell.

Fig 5A presents three examples of stationary cells. Cell I displays single patch oscillatory activator dynamics with a single patch appearing and disappearing alternately on opposite sides of the cell. This makes the shape of cell to resemble an egg with its centre oscillating along a trajectory that is almost a segment. Cell II presents rotational dynamics with the patch of activator moving around the cortex, which leads to a circular trajectory and an oval shape of the cell rotating along with the patch.

#### Persistent runner cells.

The second class of motility of simulated cells is the persistent runner. These cells have a stable pattern of activator in the cortex and move in a fixed direction. Thus, within the accuracy of our simulations, they reach a stationary state. Persistent runners appear with a plethora of attained shapes, as shown in Fig 5B: a stadium geometry (cell IV); a fan shape with broader front and contracted rear (cell V); a dumbbell shape, in which the protrusive force is trying to extend the front while surface tension is resisting the elongation (cell VI); or a keratocyte-like shape with a broad curved front and straight rear (cell VII).

#### Alternating runner cells.

The third class, alternating runner cells, is characterised by oscillatory dynamics of activator molecules in the cortex giving rise to the characteristic motion with irregular turns separated by episodes of straight motion, as exemplified in Fig 5C (cell VIII). In this case, the patch of activator does not relocate itself to the exact opposite side of the cell, but fluctuates slightly with each oscillation producing a staggered motion, as can be seen from the trajectory in Fig 5C.

This process is also reflected in the magnitude of polarity, as shown in the graph in Fig 5C: polarity drops during turn episodes when the entire cortex becomes active, and increases during run episodes. Furthermore, each run episode is accompanied by an increase in elongation of the cell body along the direction of motion. This elongation follows the peak of polarity during a given run. Once the activator patch begins to relocate, the forces along the cortex become more evenly distributed, causing the cell to contract back toward a more circular shape.

This class emerges naturally for simulations initiated with an oscillatory concentration profile with coupling parameters tuned to the crossover between stationary and persistent runners regions. However, it can also arise when simulations are initiated with a rotational profile with coupling constants at the edge of stability of rotation. In such cases, the cells typically undergo a transient phase, during which other characteristic behaviours may appear, before finally settling into alternating run.

#### Circular runner cells.

The next class is the circular runners which groups cells that move along almost perfect circle rotating around a fixed point. This motion is always accompanied with the rotation dynamics of the patch of activator in the cortex. In our simulations we distinguish two variants of this class as presented in Fig 5D. The circular runner can have either a fan shape with the elongation direction almost perpendicular to the direction of motion (cell IX in Fig 5D) or a stadium shape with the elongation roughly parallel to the cell velocity (cell X in Fig 5D). As shown in the graph of the velocity autocorrelation function in Fig 5D, fan shaped cells tend to have a larger angular velocity than the stadium-shaped cells. In the extreme case, circular runners of a stadium shape (cell X in Fig 5D) may be quite similar to some of the stationary cells (cell II in Fig 5A), with the caveat that in this case the geometric centre of a cell is sufficiently displaced to cross the initial cell-shape boundary.

#### Run-and-turn cells.

The final class observed in our simulations are run-and-turn cells. The resulting dynamics consist of periods of straight motion separated by turns. Typical example of run-and-turn cell is presented in Fig 5E (cell XI). The cell appeared as a persistent runner for the first 600 s of simulation time, after which the turning began. As the cell moves, the initial fan shape of cortex (point 1 on the trajectory and snapshot 1 in Fig 5E) becomes more and more elongated. As a result, the cell slows down, and the patch of activator spreads (point 2 in Fig 5E) and, eventually, splits into two. Then, in a short time one of the new peaks starts to dominate (point 3 in Fig 5E). Lastly, the smaller peak completely disappears (point 4 in Fig 5E), making the cell turn and reform fan shape. The whole process then repeats itself. For example, cell XI has made three turns over the simulation time.

We note that, run-and-turn cells and alternating runners have very similar trajectories. Nevertheless, the underlaying dynamics of activator is completely different. It is oscillatory for alternating runners, while being static with occasional loss of stability for run-and-turn class.

## Results: Phase diagrams and properties of the subsystems

**Fig 6:**
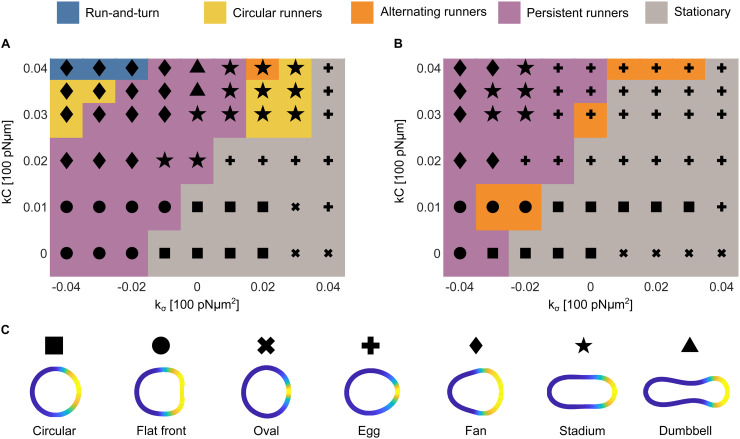
Phase diagrams. Phase diagrams for limit cycle motility phenotypes as a function of coupling strength (A) for cells initiated with a rotational concentration profile and (B) for cells initiated with an oscillatory concentration profile. Colour represents the motility class as shown above the panels, while marker represents characteristic shape of the cell. (C) Legend specifying qualitatively the characteristic shapes and patterns observed in the simulations. Quantitative analysis of simulated cells is presented in Fig 7.

**Fig 7:**
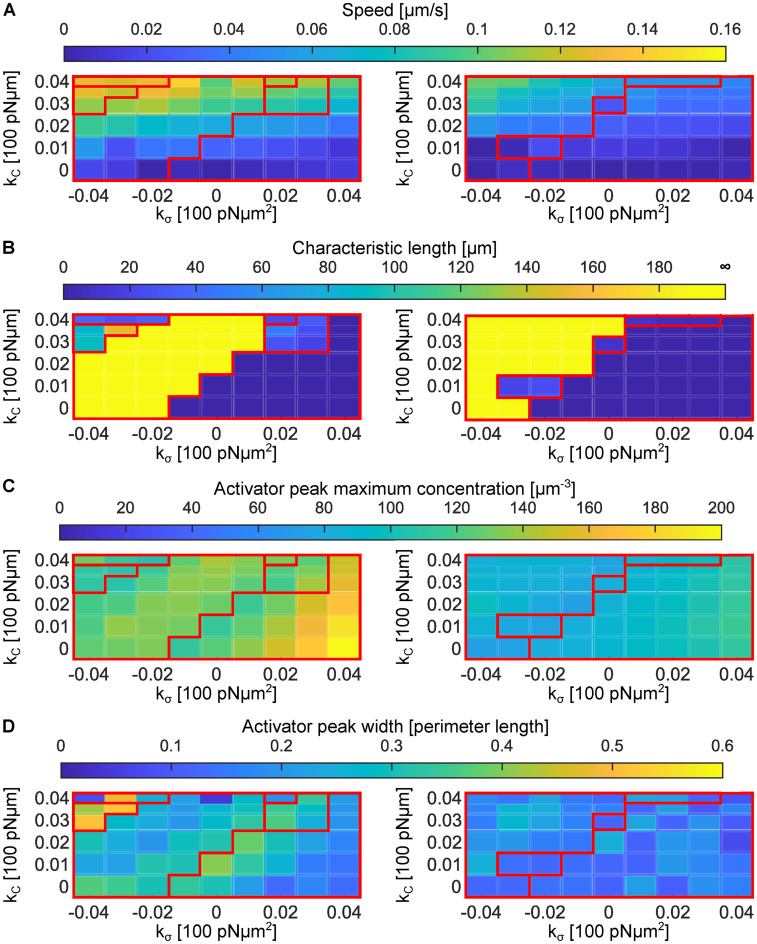
Cell characteristics as a function of cytoplasmic and cortical flow coupling constants $k_{𝐂}$ and $k_{\sigma }$. The parameters range is the same as for the phase diagram graphs in Fig 6. Red lines denote borders of regions with different motility patterns. Left panels present data for cells initiated with a rotational concentration profile, while on the right cells were initiated with an oscillatory concentration profile. The magnitude for each quantity is denoted with the colour code, as presented in the colour bar above each diagram. (A) The average speed of cell calculated for simulation time 900–1000s. (B) Characteristic length of motion. The length is defined as the circumference of the trajectory for circular runners, length of a segment between two turning points for alternating runners and run-and-turn cells. It is set to zero for stationary cells and infinite for persistent runners. (C) Activator concentration $C_{a}$ at its peak in the membrane pattern. (D) Width of the pattern of activator concentration relative to the cell circumference size. Properties of the membrane patterns shown in C and D were extracted 1 000 s after the onset of the simulation, or at the closest maximum for the oscillatory or run and turn pattern.

Analysis of previous trajectories shows that the magnitude of cytosolic and cortical flows, as well as cell velocities are primarily controlled by the strength of the active forces coupling signalling to mechanics. The dominant contributions arise from the protrusive coupling $k_{C}$ and the activator-dependent surface-tension coupling $k_{\sigma }$, the latter generating Marangoni-type stresses. To explore in more detail the effect of $k_{C}$ and $k_{\sigma }$ on the appearance of different motility modes and phenotypes, we systematically explore the phase diagram of motility modes, spanned by these parameters (Fig 6). The sensitivity of the long term dynamics at identical parameters is also explored by initiating the simulations with the circular (Fig 6A) and oscillatory (Fig 6B) profile of the RD system. Motility class and characteristic shape phenotype (Fig 6C) are determined after about 1 000 s of simulation time, when we also determine the cell velocity (Fig 7A) and persistence length (Fig 7B). This is naturally a limitation as in some rare cases development of the asymptotic cycle may take much longer than the time of our simulation.

The characteristic shapes, and associated properties of the activator distribution (width and amplitude of pattern shown in Fig 7C,D), are selected from the contour that best represents the typical morphology over the simulated interval.

### Sensitivity to the initial state of the RD system

The observed dependence of the behaviour of the system on the initial state of the simulation can be explained with two distinct effects: First, since the oscillatory and rotational initial states differ in total protein numbers, their peak amplitudes also differ; consequently, larger coupling strengths are required for cells initiated from oscillation to achieve the same dynamics as for those initiated from rotation. The second key difference lies in the concentration profiles: the oscillatory state is symmetric, providing no built-in symmetry breaking, whereas the rotational state is inherently asymmetric. This lack of initial asymmetry in the oscillatory case affects the ability of cell to express certain behaviours (*e.g.*, those that require directional bias) unless the couplings are sufficiently strong to spontaneously break this symmetry (with the help of numerical noise).

### Sensitivity to the strength of the coupling constants

Overall, the average migration speed increases monotonically with $k_{C}$. A weaker but systematic increase is also observed as $k_{\sigma }$ becomes more negative ($k_{\sigma }<0$), consistent with the dispersive flow in this regime (Fig 7A). Interestingly, this is not directly reflected in the pattern of the activator (Fig 7C,D). The intensity of the peak of the activator on the interface is directly dependent on $k_{\sigma }$, while the distribution (Fig 7D), much more strongly couples to the cell shape, and motility phenotypes, particularly in the phase diagram initiated from rotations. The combined result of these couplings of the RD system with cortical and cytoplasmic flows clearly induce very different behaviour of cells over all scales, as discussed in the following.

#### Stationary cells.

These solutions emerge when the protrusive coupling $k_{C}$ is small compared to surface tension coupling $k_{\sigma }$, and are abundant for $k_{\sigma }>0$. Stationary cells typically stay in the type of RD dynamics of the activator in which they were initialized. However, as shown in Fig 3D, increase of the coupling $k_{\sigma }$ reduces a period of observed dynamics and favours oscillations over rotations. Another interesting example of changing of RD dynamics is shown by cell III in Fig 5A, where, by increasing the number of proteins, the cell was forced into oscillation activator dynamics with two simultaneously active regions. The cell is stretched by the patches alternately in vertical and horizontal direction making its shape elliptical with only a minimal motion of its centre of geometry. However, despite being stationary, the RD system typically shows a strong peak in the activator concentration with the magnitude growing upon increasing $k_{\sigma }$ and reducing $k_{C}$, see Fig 7C.

#### Persistent runners.

These cells are equally abundant in the phase space as the stationary cells. They rely on strong protrusive forcing, combined with sufficiently large activator-dependent surface-tension coupling to maximize sustained translation. The result are two symmetric vortices in the cytoplasm flow and a stationary pattern at the moving front. Consequently, persistent runners can attain some of the highest speeds reported in Fig 7A, with a variety of shapes (Figs 5 and 6). For cells of approximately 12 µm in diameter and projected areas of 120 µm^2^, the model predicts velocities that increase with both $k_{C}$ and $\left|k_{\sigma }\right|$, under the constraint $k_{\sigma }⩽0$ and span from 0.01 to 0.16 µm/s.

This persistent translation is driven by several different stationary patterns of the activator on the membrane, which is also reflected in different shapes observed in Fig 5. Interestingly, however, the distribution of the pattern over the cell shape is relatively well preserved (Fig 7C,D).

#### Circular runners.

An interesting behaviour is observed on the crossover between stationary cells and persistent runners for $k_{\sigma }>0$ and cells initialized with the rotational state (Fig 6A). In this regime, in stadium-shape cells, the internal flows are able to move the cell, but are not strong enough to induce a persistent translation. As a result, cells become circular runners circling relatively fast (0.05–0.1 µm/s) with a relatively small radii (Fig 7B).

Above persistent runners in the phase diagram, at even larger values of $k_{C}$ and more negative $k_{\sigma }$, we find fan-shaped circular runners. They move at speeds comparable to the stadium cells but perform loops of large radii (5–80 µm for the studied range of parameters, see Fig 7B). Hence, these cells have much smaller angular velocities ($\omega =0.00125-0.02s^{-1}$). This family of solutions is also associated with the widest peaks of the activator (Fig 7D), which sustain the entire moving front of the cell.

#### Alternating runners.

This motility mode appears mostly for cells starting with an oscillatory pattern, at the crossover between stationary cells and persistent runners. Cells in this region of parameters constantly switch between being stationary and persistent runners, which makes them fall into category of alternating runners. This is a consequence of a homogenisation of the RD pattern over the cell surface or the transient creation of double oscillations (two oscillating patches). Cells in this mode exhibit speeds below 0.1 µm/s (approximately 1% of the cell size per second) and persistence lengths of 20–40 μm (about 2–4 cell lengths). Their time-averaged speed is lower than that of persistent runners because turning events necessarily involve transient slowing. In these regimes, the activator is more concentrated (small peak widths in Fig 7D), but the intensity of the peak remains moderate (Fig 7C).

#### Run-and-turn cells.

In the regime between fan-shaped circular runners and persistent runners, for large values of $k_{C}$ and for strongly negative $k_{\sigma }$ we find run-and turn cells. In this case, internal flows inside the cell become strong enough to perturb the straight motion of the cell. While larger perturbation move the stationary state into a closed trajectory, somewhat smaller couplings allow for random turns associated with the run-and-turn category. In this case, superposition of protrusive and dispersive flows makes the patch of activator on the cortex of the cell unstable. We were able to record this mobility phenotype only for cells with a rotational initial state.

## Discussion: Comparison with experiments

For complete validation of the model a direct quantitative comparison with experiments would ultimately be required. This is a difficult task due to the the multi-scale nature of the problem encapsulated also in a relatively large parameter set (see Tables A and B in S1 Appendix). Furthermore, such experiments are highly demanding as they would require simultaneous, time-resolved mapping of membrane signalling patterns, cytosolic flows, cell shape dynamics, and migration trajectories over extended periods of time, while systematically probing the phase space of motility modes. Ideally, controlled perturbations of at least one of the coupled subsystems (signalling networks, intracellular flows, cell shape, or motility) would be performed and the system response quantitatively characterised. Achieving this level of experimental control and resolution approaches current technological limits. However, there is a significant body of data where individual subsystems have been characterised for different cell types. Comparison with some of this data may be instructive to understand the role of different mechanisms acting on different scales, and the importance of their coupling. Here, the model can provide relevant insights by exploring estimates of flow velocities, cell speeds, persistence lengths, and properties of membrane patterning as shown in Fig 7.

### Persistent runners

The calculated velocities are well within the range observed experimentally for amoeboid cells migrating in uniform, cue-free environments. For example, *Dictyostelium discoideum* exhibits basal directional motility at speeds of 0.05–0.25 µm/s, with persistence over timescales of 5–15 min in the absence of external gradients [97,98]. Neutrophils, with diameters of 12–15 µm (projected areas of 110–180 µm^2^), display basal persistent motion in homogeneous conditions with typical speeds of 0.17–0.50 µm/s and persistence times of 2–8 min [99,100]. Similarly, T cells, with diameters of 7–12 µm (projected areas of 40–110 µm^2^), exhibit autonomous motion *in vivo* in lymph nodes, with typical speeds of 0.13–0.30 µm/s but relatively short persistence times [101,102]. Importantly, due to their amoeboid nature, the mechanical output of all these cells is low. For example, *Dictyostelium* and neutrophils generate traction forces on the order of 1–10 nN [103–105], while T cells operate at even lower mechanical regime, typically exerting forces that do not exceed 2 nN during autonomous migration [106]. These results agree well with the mechanical dissipation predicted in the model, which amount up to 2 nN for the parameters reported in Table A in S1 Appendix. Together, these results demonstrate our current approach very well captures this amoeboid mode of motion and points to the importance of the interplay between cortical and cytoplasmic flows.

For larger cells, such as fish epithelial keratocytes, which typically possess lengths of 15–25 µm and projected areas of 200–500 µm^2^ [107,108], the physical scale in the model must be adjusted (Table A in S1 Appendix). Accounting for a higher total number of activator molecules and appropriately increased $k_{C}$ and/or $k_{\sigma }$, our model yields cytosolic flow velocities on the order of 0.1–1 µm/s, consistent with experimental measurements of intracellular fluid dynamics yielding 0.1 µm/s or approximately 40% of the total cell migration speed [109,110]. These cells exhibit remarkably persistent migration, maintaining a stable fan-like shape for intervals typically exceeding 15 min in uniform environments [111], and generate total traction forces of approximately 10–45 nN [112,113]. While a more complex model could be considered to account for such strong contractile forces affecting the cell shape, it seems that our approach still performs reasonably well, particularly in capturing the cell shape and velocity. However, difficulties with the model are expected for large fibroblasts, with diameters 40–70 µm and projected areas 1500–4000 µm^2^, which can also display persistent migration on homogeneous 2D substrates. Typical speeds are 0.008–0.033 µm/s, persistence times range from 30–120 min, and total traction forces are of the order of 50–200 nN [114,115]. Accounting for this situation may require very large values for the dissipation coefficient β, to compensate for the simplistic model of the interaction of the cell with the substrate.

### Circular runners

In nature, circular patterns were reported for *Dictyostelium discoideum* [6] but the most stable circular trajectories were found in keratocytes [116]. This mode of motion was associated with asymmetry in myosin contractility resulting in biased retrograde actin flow and lamellipodial polymerisation slightly stronger on one side of the leading edge. The cells typically move with speeds that are a little slower but comparable to persistent runners performing circles with radii of about 25 µm and shapes exhibiting smaller aspect ratios at the inner side [116]. This is highly reminiscent of shapes and activator distributions seen in rotating fan shapes in Fig 5D, and supported by cytosolic speeds and persistence lengths as reported in Fig 7. These results show that the model spontaneously produces biologically meaningful predictions and can capture the interplay between the signalling, flows, shape and motility patterns.

### Alternating and Run-and-turn runners

Motility patters with intrinsic intermittent motility, have been observed in a number of cells including dendritic cells [117], T cells [118], or neutrophils [119]. Across these cells, basal stop-and-go dynamics emerge from a self-organizing excitable polarity network in which stochastic Rac/Ras activation drives local actin polymerization through positive feedback, while global inhibitory mechanisms (*e.g.*, myosin II contractility and membrane tension) destabilize and reset polarity. This produces spontaneous cycles of persistent runs and repolarization without external cues, which would be reminiscent of our mode of alternating runners. The statistics of run and stop times is highly dependent on the system as well as the methodology of the sampling. Hence, direct comparison with the prediction of the model should be performed on a case-by-case manner.

Similar dynamics was reported for *Dictyostelium discoideum* [97] and keratocytes [116], where frequent waddling of the cell direction was observed, presumably without a full loss of polarity. This behaviour would be more consistent with a run-and-turn mode of our model. However, without a simultaneous tracking of the cell shape, the trajectory and the changes in the patterns of the RD system, it is difficult to fully differentiate between these two modes, and further work is necessary to make this distinction.

## Conclusions

In this paper, we proposed a cross-scale mean-field theoretical framework to capture cellular locomotion for cells crawling on surfaces. By uniting intracellular signalling dynamics with spatial cytosolic flow and explicitly accounting for cortical and membrane surface flows, our model reproduces the wide range of experimentally observed cellular morphologies and motility patterns through small variations in coupling constants. Since these behaviours emerge as limit cycles, they reveal that coupling among these subsystems gives rise to intrinsically self-organised dynamics. Phase diagrams further demonstrate that the two types of coupling of the reaction–diffusion dynamics, with cytosolic flows—via protrusive force and spatially varying surface tension—are essential, along with diffusion dynamics, to recover the full repertoire of motility phenotypes observed in living cells. By systematically mapping the characteristic states of this coupled system, our work establishes a theoretical foundation that can inform and constrain future modelling and experimental efforts.

The current formulation is particularly suitable for investigating motility of cells lacking prominent contractile structures such as stress fibres, and amoeboid locomotion predominantly driven by actin polymerization [120]. However, faithfully capturing myosin-based contractility would likely require the inclusion of active stress coupling via a vector field representation, as discussed elsewhere [31,121]. Another natural extension involves the introduction of spatially variable viscosity between the external medium, the cortex, and the cytosol [122], which would enhance physical realism but demands a higher numerical resolution. Such a refinement would also allow the study of cortical ruffling phenomena arising from locally negative surface tension [123].

The current formulation and these extensions should ultimately be validated through a direct quantitative comparison with experiments, representing the next step for this framework. On a qualitative level, our model confirms indications of current state-of-the-art models and experiments [32,92,124,125] that cytosolic flow, cell shape, and RD dynamics are tightly coupled in motile cells, jointly enabling a wide range of complex behaviours. It is also consistent with the recent work that also links signalling dynamics to energetic partitioning at the cortex [126].

In conclusion, our formulation encapsulates these interacting processes and highlights the critical role of two-dimensional cortical transport and the coupling between reaction–diffusion dynamics and membrane tension. This interplay generates Marangoni flows that, together with Fickian diffusion, mediate cross-talk between the cytosolic and cortical layers. As a result, microscopic membrane patterning becomes dynamically linked to across-cell signalling and hydrodynamic feedback, jointly governing cell shape, polarity, and locomotion patterns at the mesoscopic scale.

## Supporting information

S1 Appendix Supplementary information. Description of the details of the model, its numerical implementation, the method of analysis of the results, and parameters used in the simulations together with supplementary figures and tables.
(PDF)

S1 Video Cell I. Video presenting the time evolution of cell I presented in Fig 5A.
(MP4)

S2 Video Cell II. Video presenting the time evolution of cell II presented in Fig 5A.
(MP4)

S3 Video Cell III. Video presenting the time evolution of cell III presented in Fig 5A.
(MP4)

S4 Video Cell IV. Video presenting the time evolution of cell IV presented in Fig 5B.
(MP4)

S5 Video Cell V. Video presenting the time evolution of cell V presented in Fig 5B.
(MP4)

S6 Video Cell VI. Video presenting the time evolution of cell VI presented in Fig 5B.
(MP4)

S7 Video Cell VII. Video presenting time evolution of cell VII presented in Fig 5B.
(MP4)

S8 Video Cell VIII. Video presenting time evolution of cell VIII presented in Fig 5C.
(MP4)

S9 Video Cell IX. Video presenting the time evolution of cell IX presented in Fig 5D.
(MP4)

S10 Video Cell X. Video presenting the time evolution of cell X presented in Fig 5D.
(MP4)

S11 Video Cell XI. Video presenting the time evolution of cell XI presented in Fig 5E.
(MP4)
