# Supplementary material for: Diversity in emergent cell locomotion from the coupling cytosolic and cortical Marangoni flows with reaction–diffusion dynamics
Source: PLoS Comput Biol. 2026 Apr 27;22(4):e1014216. doi: 10.1371/journal.pcbi.1014216 (PMC13148778; doi:10.1371/journal.pcbi.1014216)
Supplement: S1 Appendix — Description of the details of the model, its numerical implementation, the method of analysis of the results, and parameters used in the simulations together with supplementary figures and tables. (PDF) [file pcbi.1014216.s001.pdf]

Diversity in emergent cell locomotion from the coupling cytosolic and cortical Marangoni flows with reaction–diffusion dynamics. S1 Appendix

Blaž Ivšić,[1,2] Dorijan Vulić,[1] Igor Weber,[3] Piotr Nowakowski,[1] and Ana-Sunčana Smith[1,4,5,*]

[1] *Division of Physical Chemistry, Ruđer Bošković Institute, Zagreb, Croatia*
[2] *Centre for Advanced Laser Techniques, Institute of Physics, Zagreb, Croatia*
[3] *Division of Molecular Biology, Ruđer Bošković Institute, Zagreb, Croatia*
[4] *Faculty of Sciences, Friedrich-Alexander-Universität, Erlangen, Bavaria, Germany*
[5] *Competence Center Engineering of Advanced Materials, Friedrich-Alexander-Universität, Erlangen, Bavaria, Germany*

(Dated: April 16, 2026)

A. METHODS

1. Implementation of the model equations

To numerically solve the system of equations describing the model, one first needs to discretize the computational domain, impose suitable boundary conditions, and approximate the spatial derivatives of the velocity, level-set (LS) function, and concentration fields. Once these approximations are in place, the equations can be advanced in time. The details of this procedure is delineated in the remainder of the text.

A. Simulation grid

Model equations were discretised on the two-dimensional computational domain, which is an uniform Cartesian lattice, $[0, L_x] \times [0, L_y]$. In all simulations we adopted a square grid $\Delta x = \Delta y$. The domain is partitioned into $I - 1$ cells in the x-direction and $J - 1$ cells in the y-direction. Periodic boundary conditions are applied on all sides (Fig A). Such mesh is then topologically equivalent to the surface of a torus. Operationally, the last column of nodal values is set equal to the first column and, analogously, the last row is set equal to the first, consequently, the four corner nodes represent the same physical point. We adopt a staggered marker-and-cell (MAC) arrangement, in which scalar quantities (pressure, level-set, concentration, *etc.*) are defined at cell centres, whereas velocity components are stored on the corresponding cell faces (see Fig A). We label the face centred velocities with $V_x(i + 1/2, j)$ and $V_y(i, j + 1/2)$ and the interpolated cell centred velocities with $v_x(i, j)$ and $v_y(i, j)$.

B. Spatial discreditization

To discretize the model fields on the domain defined above, we employ a second-order central-finite-difference (CFD) scheme. Three differential operators appear in the governing equations: divergence ($\nabla\cdot$), gradient (∇), and the Laplacian (∇^2), each involving first- or second-order derivatives. Throughout the Supplementary methods, we will adopt the following notation:

- **Cell-centred scalars and vectors:** $H(i, j)$ and $\boldsymbol{H}(i, j)$ with Cartesian components $H_x(i, j)$ and $H_y(i, j)$.
- **Face-centred vectors:** $\boldsymbol{G}$ with components $G_x(i + 1/2, j)$ and $G_y(i, j + 1/2)$.

Here $i \in [1, \ldots, I]$ and $j \in [1, \ldots, J]$ index columns and rows, respectively. Depending on the variable being differentiated and the equation being discretized, several stencils are required; below we list those actually used.

a. Face-to-cell interpolation and derivatives. In the MAC layout the incompressible Navier–Stokes equation (Eq (5) in the main paper) is solved on cell faces, whereas pressure p and body force $\boldsymbol{f}$ reside at cell centres. Likewise, the level-set (Eq (1) in the main paper) and reaction-diffusion-advection equation (Eq (8) in the main paper) require

* asmith@irb.hr, ana-suncana.smith@fau.de

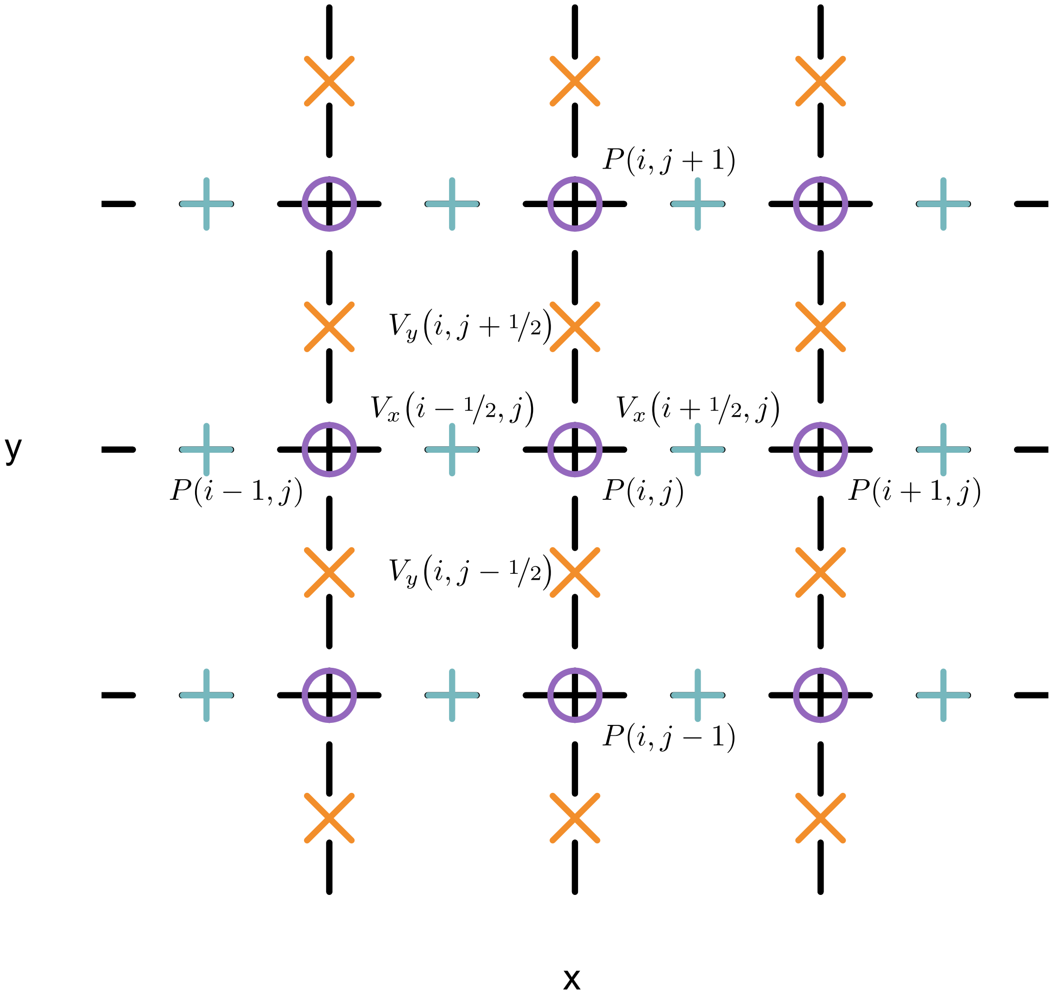

Fig A. Schematic of MAC grid with indicated shifted points at which velocity components v_x and v_y are defined. Purple circles ($\circ$) represent main grid points indexed with i and j, Orange crosses ($\times$) represent face points at which velocity v_x is defined and teal pluses ($+$) represent face points at which velocity v_y is defined.

velocities interpolated to cell centres. For a face-centred vector $\boldsymbol{G}$:

$$G_x(i,j) = \frac{G_x\left(i+1/2,j\right) + G_x\left(i-1/2,j\right)}{2}, \tag{S1a}$$

$$G_y(i,j) = \frac{G_y\left(i,j+1/2\right) + G_y\left(i,j-1/2\right)}{2}. \tag{S1b}$$

First-order derivatives at the same cell centres follow as

$$\frac{\partial G_x}{\partial x}\left(i,j\right) \approx \frac{G_x\left(i+1/2,j\right) - G_x\left(i-1/2,j\right)}{\Delta x}, \tag{S2a}$$

$$\frac{\partial G_y}{\partial y}\left(i,j\right) \approx \frac{G_y\left(i,j+1/2\right) - G_y\left(i,j-1/2\right)}{\Delta y}, \tag{S2b}$$

$$\boldsymbol{\nabla}\cdot\boldsymbol{G}\left(i,j\right) = \frac{\partial G_x}{\partial x}\left(i,j\right) + \frac{\partial G_y}{\partial y}\left(i,j\right). \tag{S2c}$$

b. Cell-to-face interpolation and derivatives. For a cell-centred scalar H:

$$H\left(i+{}^{1}\!/{}_{2},j\right)=\frac{H\left(i+1,j\right)+H\left(i,j\right)}{2},\tag{S3a}$$

$$H\left(i,j+{}^{1}\!/{}_{2}\right)=\frac{H\left(i,j+1\right)+H\left(i,j\right)}{2},\tag{S3b}$$

$$\frac{\partial H}{\partial x}\left(i+{}^{1}\!/{}_{2},j\right)\approx\frac{H\left(i+1,j\right)-H\left(i,j\right)}{\Delta x},\tag{S3c}$$

$$\frac{\partial H}{\partial y}\left(i,j+{}^{1}\!/{}_{2}\right)\approx\frac{H\left(i,j+1\right)-H\left(i,j\right)}{\Delta y}.\tag{S3d}$$

Second derivatives needed in Navier–Stokes equation (Eq (5) in the main paper) are evaluated on faces as:

$$\frac{\partial^2 G_x}{\partial x^2}(i+{}^{1}\!/{}_{2},j)=\frac{G_x(i-{}^{1}\!/{}_{2},j)-2G_x(i+{}^{1}\!/{}_{2},j)+G_x(i+{}^{3}\!/{}_{2},j)}{\Delta x^2},\tag{S4a}$$

$$\frac{\partial^2 G_y}{\partial y^2}(i,j+{}^{1}\!/{}_{2})=\frac{G_y(i,j-{}^{1}\!/{}_{2})-2G_y(i,j+{}^{1}\!/{}_{2})+G_y(i,j+{}^{3}\!/{}_{2})}{\Delta y^2}.\tag{S4b}$$

c. Cell-centred operators. At cell centres the gradient and divergence use the standard symmetric second-order stencil:

$$\frac{\partial H}{\partial x}\left(i,j\right)\approx\frac{H\left(i+1,j\right)-H\left(i-1,j\right)}{2\Delta x},\tag{S5a}$$

$$\frac{\partial H}{\partial y}\left(i,j\right)\approx\frac{H\left(i,j+1\right)-H\left(i,j-1\right)}{2\Delta y},\tag{S5b}$$

$$\boldsymbol{\nabla}\cdot\boldsymbol{H}\left(i,j\right)=\frac{\partial H_x}{\partial x}\left(i,j\right)+\frac{\partial H_y}{\partial y}\left(i,j\right),\tag{S5c}$$

$$\boldsymbol{\nabla}H\left(i,j\right)=\frac{\partial H}{\partial x}\left(i,j\right)\hat{\boldsymbol{x}}+\frac{\partial H}{\partial y}\left(i,j\right)\hat{\boldsymbol{y}},\tag{S5d}$$

where $\hat{\boldsymbol{x}}$ and $\hat{\boldsymbol{y}}$ denote unit vectors in the direction of x- and y-axis, respectively.

d. Laplacian at cell centres. Two alternative Laplacian stencils are used:

(a) *Four-point, two-step stencil* (accurate for conservative balances):

$$_1\nabla^2 H\left(i,j\right)=\frac{\partial^2 H}{\partial x^2}\left(i,j\right)+\frac{\partial^2 H}{\partial y^2}\left(i,j\right)\approx\frac{H\left(i-2,j\right)-2H\left(i,j\right)+H\left(i+2,j\right)}{4\Delta x^2}+ \\ +\frac{H\left(i,j-2\right)-2H\left(i,j\right)+H\left(i,j+2\right)}{4\Delta y^2}.\tag{S6a}$$

(b) *Nine-point isotropic stencil*:

$$_2\nabla^2 H\left(i,j\right)\approx \\ \frac{\gamma}{2\Delta x\,\Delta y}\left[H\left(i-1,j-1\right)+H\left(i-1,j+1\right)+H\left(i+1,j-1\right)+H\left(i+1,j+1\right)\right] \\ +\frac{1-\gamma}{\Delta x\,\Delta y}\left[H\left(i-1,j\right)+H\left(i+1,j\right)+H\left(i,j+1\right)+H\left(i,j-1\right)\right]+\frac{2\gamma-4}{\Delta x\,\Delta y}H\left(i,j\right),\tag{S6b}$$

where $\gamma={}^{1}\!/{}_{3}$ gives a rotationally invariant stencil [1], while $\gamma={}^{1}\!/{}_{2}$ yields the most isotropic variant [2, 3]. For the calculations of the Laplace operators of concentration profiles we used isotropic nine-point stencil ($_2\nabla^2$ with $\gamma={}^{1}\!/{}_{2}$). However, we discovered that for Laplacian of localizing potentials, one needs to use the four-point, two step stencil ($_1\nabla^2$) to conserve the mass of the protein species during simulations.

C. Temporal integration and stability conditions

To advance model equations in time we employ the explicit forward-Euler scheme, which is first-order accurate:

$$H(i,j)^{t+\Delta t} \approx H(i,j)^{t} + \frac{\mathrm{d}\,H}{\mathrm{d}\,t}^{t}(i,j)\,\Delta t, \tag{S7a}$$

$$G_x(i+1/2,j)^{t+\Delta t} \approx G_x(i+1/2,j)^{t} + \frac{\mathrm{d}\,G_x}{\mathrm{d}\,t}^{t}(i+1/2,j)\,\Delta t, \tag{S7b}$$

$$G_y(i,j+1/2)^{t+\Delta t} \approx G_y(i,j+1/2)^{t} + \frac{\mathrm{d}\,G_y}{\mathrm{d}\,t}^{t}(i,j+1/2)\,\Delta t. \tag{S7c}$$

When combined with the second-order central-difference spatial discretization, forward-Euler renders the pure advection terms *linearly unstable*. Nonetheless we take care to satisfy von Neumann condition throughout our simulations with suitable parameter choices:

$$\Delta t \leqslant \min\left(\frac{h^2}{4D_{\max}}, \frac{h^2}{4\nu}, \frac{1}{k_{\max}}\right), \tag{S8}$$

where $h = \min(\Delta x, \Delta y)$ is the smaller grid spacing, ν is the kinematic viscosity, and $D_{\max}$ and $k_{\max}$ are the largest diffusion coefficient and reaction rate, respectively, in the RDA subsystem. Within the parameter ranges explored, the bound is dominated by the bulk substrate diffusivity D_{s}.

D. Incompressible Stokes-pressure algorithm

When solving the Navier–Stokes system the velocity field must remain divergence-free. We adopted standard procedure for solving Poisson equation for the pressure which enforces this constraint [4, 5]. Taking the divergence of the time-discretised momentum equation yields:

$$\frac{\boldsymbol{\nabla}\cdot\boldsymbol{V}^{t+\Delta t} - \boldsymbol{\nabla}\cdot\boldsymbol{V}^{t}}{\Delta t} = -\frac{1}{\rho}\nabla^2 p^{t+\Delta t} + \nu\nabla^2\left(\boldsymbol{\nabla}\cdot\boldsymbol{V}^{t}\right) + \boldsymbol{\nabla}\cdot\boldsymbol{f}^{t}, \tag{S9}$$

where ρ is the fluid density and ν the kinematic viscosity. By imposing the incompressibility condition $\boldsymbol{\nabla}\cdot\boldsymbol{V}^{t+\Delta t} = 0$ we arrive at the Poisson problem:

$$\nabla^2 p^{t+\Delta t}(i,j) = \rho\boldsymbol{\nabla}\cdot\boldsymbol{f}^{t}(i,j) + \rho\nu\nabla^2\left(\boldsymbol{\nabla}\cdot\boldsymbol{V}^{t}\right)(i,j) + \frac{\rho}{\Delta t}\left(\boldsymbol{\nabla}\cdot\boldsymbol{V}^{t}\right)(i,j) \equiv Z(i,j). \tag{S10}$$

Thus any divergence introduced at step t is projected out in the pressure update at $t + \Delta t$.

We solve (S10) with a classic Jacobi iteration. On a uniform grid ($\Delta x = \Delta y = h$) the five-point stencil reads $\nabla^2 p_{i,j} = (p_{i+1,j} + p_{i-1,j} + p_{i,j+1} + p_{i,j-1} - 4p_{i,j})/h^2$. Rearranging for $p_{i,j}$ gives the Jacobi update:

$$p^{(k+1)}(i,j) = \frac{1}{4}\left[p^{(k)}(i+1,j) + p^{(k)}(i-1,j) + p^{(k)}(i,j+1) + p^{(k)}(i,j-1) - h^2 Z(i,j)\right], \tag{S11}$$

where the superscript (k) denotes the iteration count. Iteration proceeds until either a prescribed maximum number of sweeps is reached or the root-mean-square change between successive iterates falls below a specified tolerance (`pTOL` in Table C). We conclude the operation by normalizing the pressure by subtracting its spatial mean so that $\langle p \rangle = 0$.

E. Level-set re-initialisation algorithm

The level-set field ϕ must be systematically reinitialised so that it retains the desired hyperbolic tangent profile. Reinitialisation is performed by marching the auxiliary equation:

$$\frac{\partial\phi}{\partial\tau} = (1 - 2\phi)\left[\epsilon\,|\boldsymbol{\nabla}\phi| - \phi(1-\phi)\right], \tag{S12}$$

in a pseudo-time τ. Above equation describes conservative reinitialisation scheme proposed by Parameswaran and Mandal [6]. Same authors propose a convenient way to measure the deviation from the target profile:

$$|\boldsymbol{\nabla}\phi| = \frac{\phi}{\epsilon}(1-\phi), \tag{S13}$$

which is satisfied when the level-set function has a desired shape. At the start of every physical time step, we test all grid nodes (i, j):

$$\left| \epsilon \left| \boldsymbol{\nabla}\phi \right| (i,j) - \phi\,(i,j)\,[1-\phi\,(i,j)] \right| < \mathtt{lsTOL}, \qquad \left| \boldsymbol{\nabla}\phi \right| (i,j) = \sqrt{(\partial_x \phi)^2 + (\partial_y \phi)^2}, \tag{S14}$$

where `lsTOL` is a desired tolerance and the discrete derivatives $\partial_x \phi, \partial_y \phi$ are evaluated with Eqs. (S5a) and (S5b). If the condition (S14) is violated at any point (i, j), we advance the reinitialisation Eq (S12) forward by one pseudo-time step $\Delta\tau$ and retest. The loop terminates once the condition is true everywhere. In practice we find that choosing[1] $\Delta\tau \approx 100\,\Delta t$ allows the procedure to converge within one or two pseudo-time iterations, and that reinitialising once every ~ 10 physical steps is mostly sufficient.

F. *Simulation workflow*

Fig B sketches the logical flow of one simulation step, summarised here for clarity:

1. **Level-set check and (optional) reinitialisation.** Test condition (S14); if violated at any node, advance the reinitialisation Eq (S12) in pseudo-time.
2. **Auxiliary-field update.**
   - Compute $\boldsymbol{\nabla}\phi$, $\nabla^2\phi$, curvature κ, interface normals $\hat{\boldsymbol{n}}$, and cell-centred velocity $\boldsymbol{v}\,(i, j)$.
   - Evaluate divergence, gradient, and Laplacian operators required by the governing equations on the appropriate grids.
3. **Gradient cut-off filter.** If $\left|\boldsymbol{\nabla}\phi\right|(i, j) <$ `gCO` at a node, set $\boldsymbol{\nabla}\phi$, κ, and $\hat{\boldsymbol{n}}$ to zero there. The threshold `gCO` is tuned so that the numerical noise remains bounded on the time scales of interest.
4. **Computation of body force.** Assemble the body-force density $\boldsymbol{f}$ from membrane tension, active stresses, and localisation potentials.
5. **Pressure projection.** Solve the Poisson problem (S10) by Jacobi iteration, terminating when either the root-mean-square update falls below `pTOL` or a maximum of `maxIter` sweeps is reached. Subtract the domain-average pressure to fix the gauge.
6. **Localisation potentials and surface–bulk coupling.** Evaluate the surface- and bulk-potential fields; if $|\phi| <$ `gCO` or $|\boldsymbol{\nabla}\phi| <$ `gCO` at a node, clamp the potential to its far-field value to avoid division by small numbers.
7. **Time integration of all fields** Advance $\boldsymbol{V}$, ϕ, and the concentrations $C_\mathrm{s}, C_\mathrm{a}, C_\mathrm{in}, C_\mathrm{c}$ by one physical time step Δt using the forward-Euler update (S7).

2. Software implementation

The complete code used to obtain the results presented in the main paper is available online at: `https://github.com/blejzara/CellRDA-LS`.

Here we document the main properties of the implementation.

Code is written in ISO C99 and consists of two translation units:

- `main.c` — reads the `.json` parameter file, loads the initial NetCDF snapshot, allocates arrays, and sets run-time options;
- `simulation.c` — contains the time-marching loop, numerical stencils, Jacobi pressure solver, and I/O wrappers.

A. *Dependencies*

Two external libraries are required, both distributed under permissive open-source licences:

1. **netCDF-C**[2]—scientific array I/O; the Debian/Ubuntu package `libnetcdf-dev` or the `conda-forge` recipe `netcdf-c` installs headers and shared objects automatically. Compilation is entirely optional at run-time, so the library can be linked statically for portability.
2. **cJSON**[3]—ultra-lightweight JSON parser used to read `input.json`. The repository provides a single `cJSON.c`/`cJSON.h` pair that can be dropped into the project and compiled together with the simulation code.

---

[1] See also the discussion in [6].
[2] `https://github.com/Unidata/netcdf-c`
[3] `https://github.com/DaveGamble/cJSON`

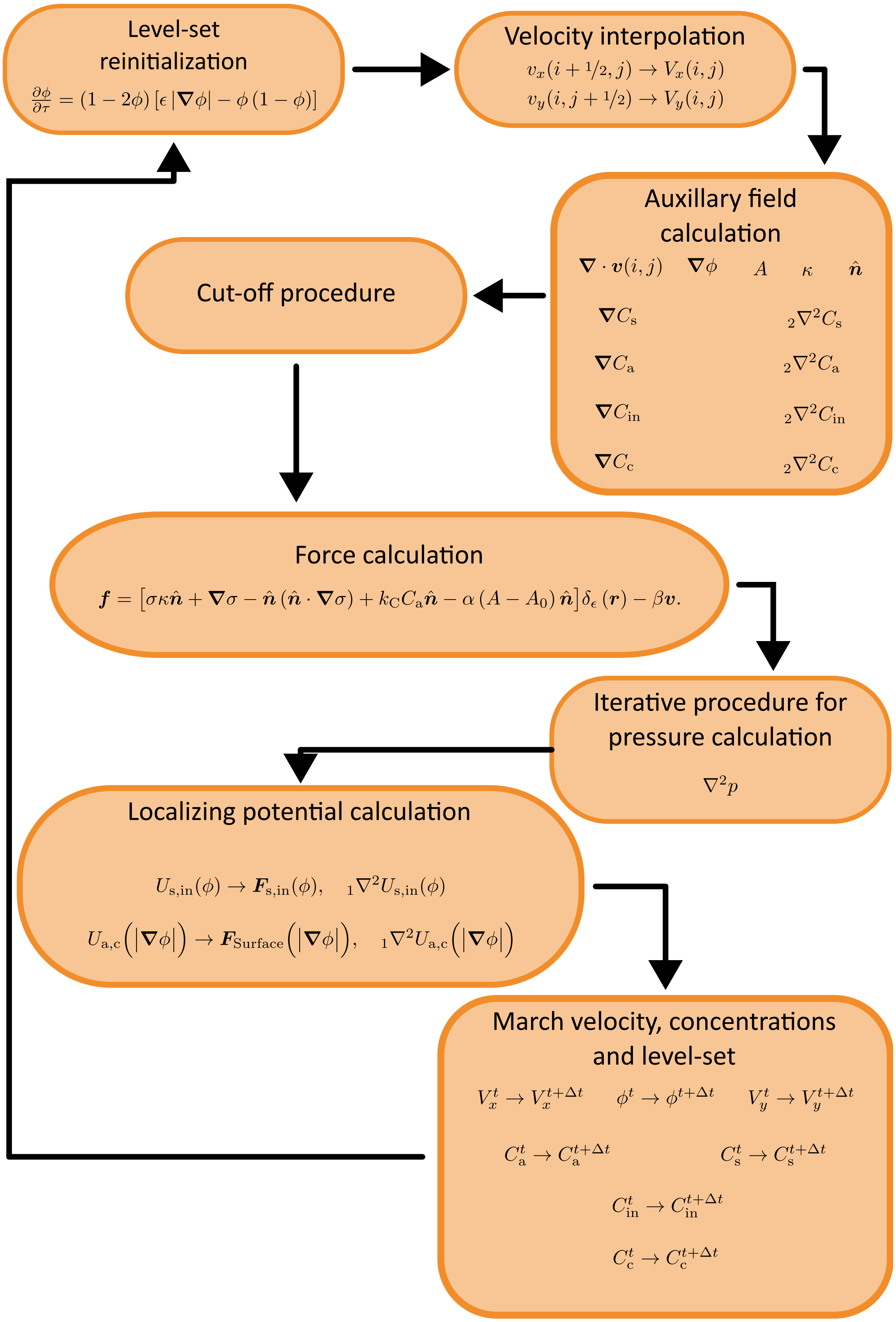

Fig B. Schematic representation of the simulation work flow.

All remaining functionality relies solely on the ISO `C99` standard library. For Windows users without a native Unix-like environment, we recommend MinGW-w64 together with the `conda-forge` channel, which supplies pre-built netCDF binaries compatible with MinGW.

B. Code architecture

Translation units and entry points:

- `main.c` is the sole executable. It parses the `input.json` parameter file with `cJSON`, loads NetCDF snapshot as initial conditions, allocates all run-time arrays via the `ALLOC_ARRAY` macro (a thin wrapper around `calloc`), and opens the output NetCDF file and binary tracer files. Command-line arguments allow batch submission on a cluster or interactive runs on a workstation.
- `simulation.c` exports a single function `simulation()` that advances the solution by invoking two high-level kernels: `PrepStep()` prepares all auxiliary fields and solves the Poisson problem, while `Step()` performs the forward-Euler update of velocities, level-set, and reaction–diffusion fields.

The main advance routine organises the simulation as a double loop. Between two output times only two copies of every field are kept in memory (current and previous) so the working-set size scales linearly with grid area. At the end of an outer cycle the current state is flushed to NetCDF, and, if enabled, tracer positions are appended to binary files. All arrays are stored in 1-D row-major buffers to maximise spatial locality.

The project targets the ISO `C99` standard, which the code requires for features such as `//` comments and inline functions. A minimal GNU the recommended flags are `-std=c99 -Wall -O3 -lnetcdf -lm`. The code has been tested with GCC 13 and Clang 17 on both Linux and Windows/MinGW-w64.

3. Analysis of solutions

With the solution NetCDF file obtained, we imported it into MATLAB R2023b [7] (build 23.2.0.2409890 Update 3) and generated `.tif` files of level-set function and activator “a” profiles. These files were then analysed in FIJI[4] [8] using the open-source QuimP plugin (version 19.08.01) [9]. Procedure of quantitative image analysis consisted of segmentation with BOA module, frame mapping with ECMM and finally extraction of concentration profile with ANA module. All other BOA settings were left at their default values except for `Node spacing` which was increased to 8 and `Final shrink` which was adjusted to 5. During the analysis with ANA module, the cortex width was set equal to parameter ϵ used in simulation.

The node- and frame-resolved data stored in the QP files were converted to MATLAB matrices (MATLAB R2023b [7]) with the unmodified conversion scripts supplied by the QuimP authors. The resulting datasets were saved as `.mat` files for downstream statistical analysis and visualisation. For transparency and reproducibility, the MATLAB routines (`convertQP2Mat.m`, `assembleAnaMat.m`, etc.) can be obtained from the supplementary repository of the project at `warwick.ac.uk/.../quimp/test_data/`[5]. Videos of simulated cells are available on Zenodo, `DOI: 10.5281/zenodo.17466622`.

Finally, Quimp generated `.mat` files were once again loaded into MATLAB (MATLAB R2023b [7]) where trajectory and contour concentration profiles were extracted and additional cell metrics calculated. This procedure was adopted to enable a direct comparison of simulations with experimentally imaged cells that will be analysed in the same way.

In the following, we present mathematical formulation of the metrics used.

A. Elongation

Moment invariants provide compact, rotation- and scale-independent descriptors of cell shape. Following Dunn & Brown [10] and Teague [11], we computed raw, central and normalised central moments of the discrete outline. From this moments one can extract parameters of equimomental elipse such as orientation and semi-axes a and b. Following Dunn & Brown, log-transformed descriptor was used:

$$E = \log_2 \left(\frac{a}{b} \right), \tag{S15}$$

which measures elongation and equals zero for a circle.

[4] ImageJ, version 1.54p

[5] Direct download of the routine bundle: `https://warwick.ac.uk/fac/sci/dcs/people/till_bretschneider/quimp/test_data/quimp11_matlab.zip`.

B. Intensity signal extraction and polarity calculation

Fluorescence profiles exported by the ANA module were resampled to the common $N = 100$ arc-length grid with `interp1(..., 'spline')`, and smoothed with `smoothdata(..., 'gaussian')` (spatial window = 5 nodes, temporal window = 10 frames). Let $I(s,t)$ denote the smoothed intensity matrix (space index $s \in [0,1]$, frame index t). Extracted intensity was then used for visualizations and polarity calculation.

We define polarity vector as weighted average radial position with respect to the centroid:

$$\boldsymbol{P}(t) = \int_0^1 I(s,t)\boldsymbol{r}(s)\,\mathrm{d}\,s \bigg/ \int_0^1 I(s,t)\,\mathrm{d}\,s, \tag{S16}$$

were $\boldsymbol{r}$ is radial vector from centroid to the position on the contour. In discrete terms used in analysis the above relation can be written as:

$$\boldsymbol{P}(t) = \sum_s I(s,t)\boldsymbol{r}(s) \Big/ \Big[\sqrt{a(t)b(t)}\sum_s I(s,t)\Big]. \tag{S17}$$

The direction of polarity vector marks the mean position of the intensity profile, which for single peak relates to its mean position. The magnitude of the polarity on the other hand relays the information how far away from the centroid is the active region on the cortex. We normalize this value with characteristic cell length $\sqrt{ab}$ to obtain dimensionless vector quantity. Where a and b are semi axes of the Legendre ellipse fitted to the contour of the cell.

C. Velocity autocorrelation function

Step length of the trajectory of a cell was calculated as difference between centroid positions in subsequent steps. To obtain velocity this value was further divided by frame interval. As before, `atan2` was used to provide the velocity angle with respect to x axis. To provide a measure of how the velocity vectors are correlated over time, velocity autocorrelation function (VACF) was determined using the following formula

$$\chi(t) = \frac{(N-t)\sum_{i=1}^{N-t} \boldsymbol{v}_i \cdot \boldsymbol{v}_{i+t} - \left(\sum_{i=1}^{N-t} \boldsymbol{v}_i\right) \cdot \left(\sum_{i=1}^{N-t} \boldsymbol{v}_{i+t}\right)}{\sqrt{\left[(N-t)\sum_{i=1}^{N-t} \|\boldsymbol{v}_i\|^2 - \left\|\sum_{i=1}^{N-t} \boldsymbol{v}_i\right\|^2\right] \cdot \left[(N-t)\sum_{i=1}^{N-t} \|\boldsymbol{v}_{i+t}\|^2 - \left\|\sum_{i=1}^{N-t} \boldsymbol{v}_{i+t}\right\|^2\right]}}. \tag{S18}$$

Here, $\boldsymbol{v}_i$ and $\boldsymbol{v}_{i+t}$ represent the velocity vectors at time steps i and $i+t$, respectively, N is the total number of frames, and t is the time lag between frames. The numerator measures the covariance between velocity vectors separated by a lag t, while the denominator normalizes the autocorrelation by the variances of the velocity vectors at the two time points. This ensures that $\chi(t)$ is dimensionless and lies between -1 and 1, where values close to 1 indicate strong positive correlation, and values near -1 indicate strong negative correlation.

D. Flow extraction

To generate the figures of flow in the main text, raw velocity data obtained during simulation was loaded from NetCDF file into MATLAB [7]. Velocity profile was first interpolated to cell centres as explained in Section A 1 B. Then, cell centre-of-mass velocity was determined by averaging the position of the LS function. The code saves LS profile at the specified times T, as well as a time step before $T - \mathrm{d}\,t$. This allows for calculation of cell centre-of-mass velocity at the resolution of the simulation.

Te obtained cell velocity was then subtracted from the velocity profile of the underling fluid to obtain velocity profile in the reference frame of the cell. To isolate only cortex and cytosolic flows, mask based on values of LS function and magnitude of its gradient were used. For cytosolic flow, velocity was plotted only at points where LS function value exceeded 0.825, while for cortex flow the velocities were plotted only at points where magnitude of gradient exceeded value of 0.25.

Table A. Physical parameters of the model that can be directly measured experimentally. For each parameter, we present its name, unit, symbol, value or range used in numerical simulations, and range of available experimental measurements with references. We use symbols L, T, and F to denote units of length, time, and force, respectively. The physical value was calculated using L = 1 µm, T = 1 s, and F = 100 pN, and it can be rescaled by using different values of base units.

**Parameter**	**Unit**	**Symbol**	**Simulation value/range**	**Physical value/range**	**Experimental range**	**Reference**
Dynamic viscosity	$\mathrm{FT/L^2}$	ν	1	100 Pa s	10 – 350 Pa s	[12]
Resting surface tension	F/L	σ_0	5	0.5 nN/µm	0.2 – 1.4 nN/µm	[13]
Cell area	$\mathrm{L^2}$	A_0	120	120 µm^2	20 – 400 µm^2	[14]
**Diffusion coefficients**						
substrate	$\mathrm{L^2/T}$	D_s	34	34 µm^2/s	3 – 24 µm^2/s	[15][a][16][b][17][c]
activator	$\mathrm{L^2/T}$	D_a	0.001 – 1	0.001 – 1 µm^2/s	0.1 – 1.1 µm^2/s	[18][d][19][e][20][f]
inhibitor	$\mathrm{L^2/T}$	D_in	8	8 µm^2/s	6 – 13 µm^2/s	[21][g][22][h]
complex	$\mathrm{L^2/T}$	D_c	0.001 – 1	0.001 – 1 µm^2/s	0.04 – 0.16 µm^2/s	[19][i][20][j]
**Reaction rates**						All reaction rates are based on [23, 24]
activator attachment	1/T	k_1	0.19	0.19 s^{-1}		
activator autocatalytic attachment	$\mathrm{L^3/T}$	k_{11}	0.027	0.027 µm^3/s		
catalytic complex attachment	$\mathrm{L^3/T}$	k_{12}	0.0045	0.0045 µm^3/s		
complex association	$\mathrm{L^3/T}$	k_2	4.5	4.5 µm^3/s		
complex dissociation	1/T	k_3	0.15	0.15 s^{-1}		
activator saturation concentration	$\mathrm{1/L^3}$	$C_\mathrm{a}^\mathrm{MAX}$	200	200 µm^{-3}		
Total species 1 molecules (a + s + c)	1	N_1	2000 – 4000	—		
Total species 2 molecules (in + c)	1	N_2	500 – 1500	—		

[a] GFP in *Dictyostelium discoideum.*
[b] Ran in HeLa cells.
[c] Cdc42 in *Saccharomyces cerevisiae.*
[d] Ras1-GDP in *Schizosaccharomyces pombe.*
[e] Rac1 mutants fast diffusion in MCF7 cells.
[f] H-Ras fast diffusion in mouse cells.
[g] ARFGAP1 in HeLa cells.
[h] GAP1 in HEK-293 cells.
[i] Rac1 mutants slow diffusion in MCF7 cells.
[j] H-Ras slow diffusion in mouse cells.

Table B. Physical parameters of the model that cannot be related directly to measurable quantities. They represent simplification of more complex physical processes, not included in the model. The meaning of each column is the same as in Table A.

**Parameter**	**Unit**	**Symbol**	**Simulation value/range**	**Physical value/range**
Surface-tension coupling	$\mathrm{F\,L^2}$	k_σ	−0.06 – 0.06	−6 – 6 pN µm^2
Protrusive-force coupling	F L	k_C	0 – 0.06	0 – 6 pN µm
Dissipation coefficient	$\mathrm{F\,T/L^4}$	β	1	100 pN s/µm^4
Area-constraint coefficient	$\mathrm{F/L^4}$	α	0.1	10 pN/µm^4

Table C. Parameters of numerical implementation of the model. The meaning of each column is the same as in Table A.

**Parameter**	**Unit**	**Symbol**	**Simulation value/range**
Domain size	L	L_x, L_y	15 – 30
Grid spacing	L	$\Delta x, \Delta y$	0.12
Time step	T	Δt	5×10^{-5}
Gradient threshold	1	`gCO`	1×10^{-3}
Level-set tolerance	1	`lsTOL`	1×10^{-3}
Pressure tolerance	1	`pTOL`	1×10^{-5}
Maximum Jacobi iterations	1	`maxIter`	6×10^4
Interface thickness	L	ϵ	1.2
Density	$\mathrm{F\,T^2/L^4}$	ρ	1

Table D. Variable parameters used in the simulations

**Cell**	L_x, L_y	k_σ	k_C	D_a	D_c	N_1	N_2
Fig 3	28	0	0.04	0.3	0.1	2400	700
Fig 4 A	28	0.03	0	0.3	0.1	2400	700
Fig 4 B	28	−0.03	0	0.3	0.1	2400	700
Fig 4 C	28	−0.03	0	0.3	0.1	2400	700
Fig 4 D	28	−0.02 – 0.05	0	0.3	0.1	2400	700
Fig 5 Cell I	28	0.04	0.02	0.3	0.1	4000	1200
Fig 5 Cell II	28	0.02	0.02	0.3	0.1	4000	1200
Fig 5 Cell III	28	0.04	0.04	0.3	0.1	3600	600
Fig 5 Cell IV	28	0	0.03	0.3	0.1	4000	1200
Fig 5 Cell V	28	−0.03	0.02	0.3	0.1	4000	1200
Fig 5 Cell VI	28	0	0.04	0.3	0.1	4000	1200
Fig 5 Cell VII	28	−0.04	0.04	0.3	0.1	2400	700
Fig 5 Cell VIII	28	0.01	0.05	0.3	0.1	2400	700
Fig 5 Cell IX	28	−0.04	0.03	0.3	0.1	4000	1200
Fig 5 Cell X	28	0.02	0.03	0.3	0.1	4000	1200
Fig 5 Cell XI	28	−0.02	0.04	0.3	0.1	4000	1200
Fig 6 A	28	−0.04 – 0.04	−0.04 – 0.04	0.3	0.1	4000	1200
Fig 6 B	28	−0.04 – 0.04	−0.04 – 0.04	0.3	0.1	2400	700
Fig C Oscillation	25	0	0	0.3	0.1	2400	700
Fig C Rotation	25	0	0	0.3	0.1	4000	1200
Fig C Static polarization	25	0	0	0.3	0.6	2400	700
Fig D Oscillation	28	0	0	0.3	0.1	2400	700
Fig D Rotation	28	0	0	0.3	0.1	4000	1200
Fig D Static polarization	28	0	0	0.3	0.6	2400	700
Fig E A	25	0	0	0.01 – 1	0.01 – 1	4000	1200
Fig E B	25	0	0	0.01 – 1	0.01 – 1	2400	700

B. PARAMETERS OF THE MODEL

The numerical values of the parameters used in our simulations are listed in Tables A–C, and D. To simplify the possible adaptation of our model to various cases, in Tables A–C we also present the units of each quantity. The exact physical values, presented in all graphs, were calculated by assuming 1 µm, 1 s, and 100 pN as units of length, time, and force, respectively.

When comparing the parameter values used in our simulation with experimentally measured quantities, two important considerations must be taken into account. First, our model is formulated in two dimensions, which means that any comparison with experimental data requires the assignment of an effective thickness to the corresponding three-dimensional slice. In this work, we assume this thickness to be 1 µm. Second, our diffuse-interface formulation introduces an additional effect. Since the interface is distributed around the 0.5 level of the LS function ϕ in the strip of the thickness given by the parameter ϵ, quantities such as forces, membrane concentrations, and reaction rates must be integrated in the direction normal to the interface in order to recover values corresponding to experimentally observable quantities on the cell membrane. This integration yields an ϵ-dependent factor, which also modifies the corresponding physical units. We note that units presented in the figures in the main paper correspond to the ones listed in these tables. In particular, this implies that interface concentration values are presented in μm^{-3}, which can be related to effective two-dimensional membrane concentration by integrating across the diffuse interface.

For kinematic viscosity ν, surface tension σ_0, and projected cell area A_0 the experimental measurements are available in the literature—we used the values of the same order of magnitude, see Table A.

The diffusion coefficients and reaction constants were adopted from previous works [24, 25]. In this earlier research, the signalling network of *Dictyostelium discoideum* was reduced to only four reaction species, recovering the experimentally-observed patterns on stationary cells. The reaction rates used in this manuscript were adjusted to reproduce the phase diagram reported therein, as discussed in detail in Section C. This required rescaling of reaction-related parameters from the earlier model to account for the spreading of the interface introduced in the present diffuse-interface formulation. Diffusion values here remain consistent with the experimentally measured ranges reported for GTPases and their regulatory proteins.

The remaining physical parameters, such as the couplings k_σ and k_C (which also scale with the width of the interface ϵ), or dissipation coefficient β are effective parameters integrating a range of microscopic properties. These parameters were treated as open and adjusted to provide reasonable speeds of the cytoplasmic streaming and total mechanical

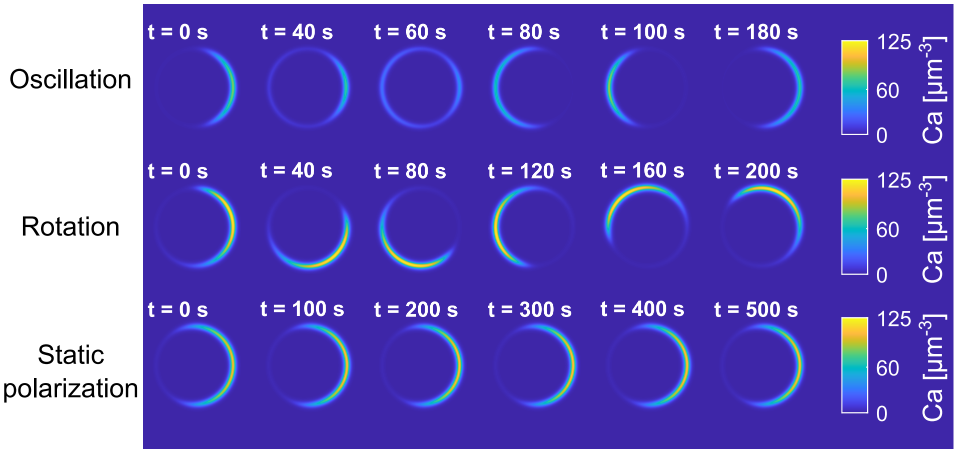

Fig C. Evolution of activator concentration C_a on a stationary circular cell. Within our model, three distinct patterns emerge: **oscillation** (top row), **rotation** (middle row), and **static polarization** (bottom row). Colour represents concentration, with colour bars shown on the right. Simulation time for each snapshot is displayed above the frame. **Oscillation** refers to two antipodal peaks of C_a that manifest alternately over time. **Rotation** describes a single concentration peak that continuously travels around the cortex (clockwise in this case). **Static polarization** is characterized by a stable, non-uniform concentration profile that does not change in time.

footprint of the cell, which can be experimentally verified, as discussed in the main text.

Finally, some of the parameters, listed in the Table C, are introduced by our numerical algorithm and have no physical meaning. For them, we have again used the trial and error method, making sure that the stability conditions of our numerical routines are satisfied. This includes the density ρ in our simulations, which was artificially upscaled to increase the time step of the simulation, while keeping the overall dynamics overdamped.

C. PROPERTIES OF REACTION-DIFFUSION MODEL

Here we will discuss the reaction-diffusion (RD) model in more detail. We aim to explain the technical details and the behaviour of solutions of our model on stationary domains without underlying advective flows and subsequent shape changes. In the main text we will refer to the canonical solutions of the model presented here flowing a naming scheme introduced bellow. Although the conditioning of RD dynamics is well documented in literature [23, 26, 27], we present it here for the sake of completeness.

1. Reaction–diffusion dynamics on circular geometry

We started our study by running preliminary simulations in which a simplified model was used—there was no advancement of the velocity field $\boldsymbol{v}$ and the LS function ϕ (using Navier–Stokes equation, Eq (5) in the main text; and LS equation, Eq (1) in the main text; respectively). This way, the initial condition with no flow and circular shape of cell was preserved during the whole simulation time, and only RD dynamics was present.

In canonical RD systems that combine activator-inhibitor, activator-substrate, and mass-conservation properties, three characteristic activator dynamics are typically observed. The simplest of these is static polarization, which can emerge via two distinct mechanisms: the first described by Turing [28] and the second, specific to mass-conserved systems, introduced by Mori *et al.* [29]. The other two patterns are characterized by a shifting or travelling polarization patch, as introduced by Gierer and Meinhardt [30, 31]. All of the above dynamics can appear with any number of high- and low-concentration zones, depending on the system size, geometry, and total protein number. In this section, we restrict our study to single-peak patterns, for clarity. These three limit-cycle patterns had been successfully reproduced by our simplified model, we present them in Fig C.

In the first row of Fig C, we observe how the concentration of the activator C_a evolves over time under the **oscillation** pattern. Here, the high-concentration patch alternates between opposite sides of the cortex, resembling a standing wave. As with all standing waves, this pattern can be thought of as the superposition of two waves travelling in opposite directions. In this case, the constituent waves need not be single-frequency sine waves; rather, they can be wave packets formed by the superposition of multiple frequency components. This viewpoint can be useful for

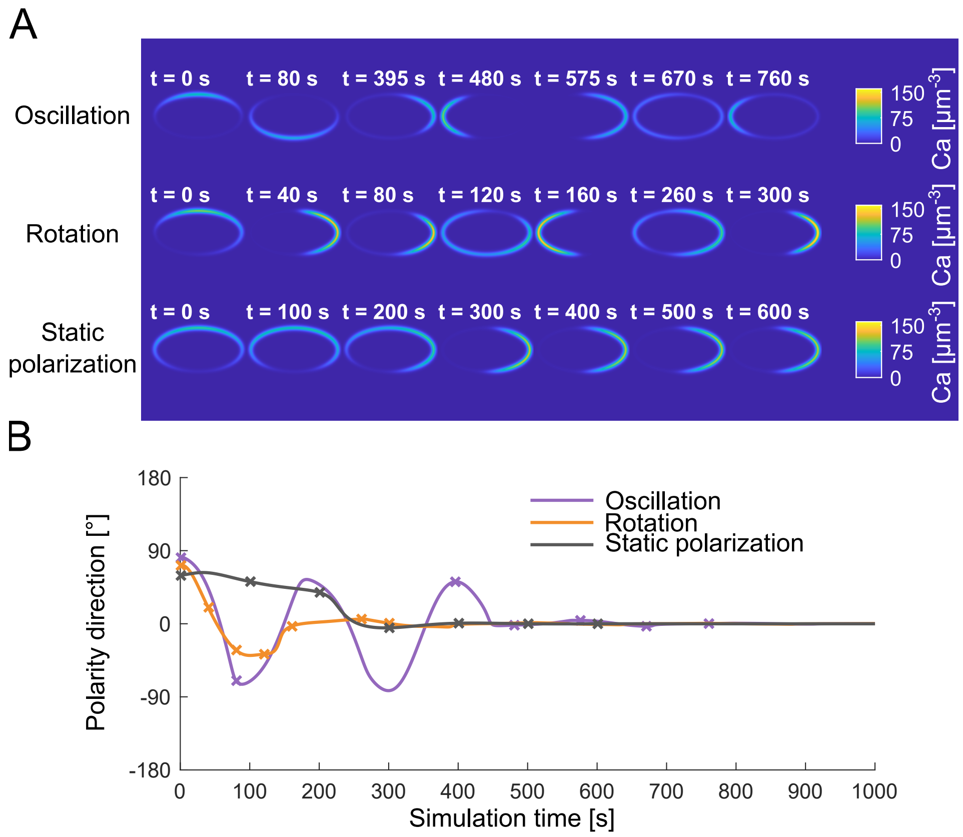

Fig D. Aligning of the dynamics of activator along the long axis of the elliptical cell. (A) Same three patterns as in Fig C, initiated on an elongated geometry (axis ratio ~ 2). Colour represents concentration C_{a}, with colour bars shown on the right. Simulation time t for each snapshot is displayed above the frame. All three patterns align with the long axis of the ellipse, with the rotation pattern transforming into oscillation. (B) Polarity direction versus simulation time for each simulation in Panel A. The angle is given in degrees, with zero defined by the positive x-axis in the simulations. Crosses on each curve mark the frames shown in Panel A. The alignment of patch along the long axis of cell is the fastest for rotation dynamics and the slowest for oscillations.

conceptualising the underlying dynamics. The oscillation period in this example is approximately 190 s, although it can be rescaled by adjusting the reaction and diffusion coefficients in the model. In terms of linear stability analysis, this pattern corresponds to a symmetric Hopf-type solution, where “symmetric” refers to each wave component having a counterpart travelling in the opposite direction.

The next pattern, which we refer to as **rotation**, is characterized by a single wave travelling around the cell cortex. Importantly, the wave does not simply diffuse around the cortex; rather, it is continuously degraded at its trailing edge and regenerated at its leading edge. As in the previous case, the high activator concentration (C_{a}) patch is not a simple sine wave but instead a wave packet, with a much steeper front than back. This asymmetric profile is characteristic of bifurcation dynamics. The period of the rotation shown here is approximately 220 s, although, as with oscillation, it can be rescaled by adjusting the reaction and diffusion coefficients. In terms of linear stability analysis, this behaviour corresponds to an asymmetric Hopf-type bifurcation.

The final pattern, which we refer to as **static polarization**, is characterized by a limit cycle in which the cell cortex remains non-uniformly polarized. In Fig C, we observe that a region of high activator concentration remains fixed on the right side of the cell for approximately 500 s of simulation time. In the literature, two distinct mechanisms for the formation of static polarization are described. The first, introduced by Turing [28], arises when diffusion and reaction processes balance, such that degradation and production (attachment) rates are equal. The second mechanism, specific to mass-conserved systems and first described by Mori *et al.* [29], occurs when polarization depletes the pool of available protein molecules, thereby stabilizing the pattern. The example shown in the last row of Fig C can be considered a Turing-type pattern. A detailed proof is beyond the scope of this paper, and the reader is referred to the work of Šoštar *et al.* [23, 25] for further discussion.

The three patterns presented above demonstrate that canonical solutions of RD systems emerge naturally within our model. The snapshots of the concentration profiles representing oscillation and rotation, obtained as the limit

cycle solutions presented above, were used as the initial concentration profiles for all of our full-model simulations. We refer to them as *oscillation* and *rotation initial patterns*.

2. Shape effects on reaction–diffusion dynamics

The effect of domain geometry on RD systems has been well documented in both numerical simulations and live-cell experiments [26, 27, 32, 33]. Alignment with the long axis occurs because the characteristic length scales of the system are closely linked to the transport processes within the cell. For example, elongating the cell changes the ratio between the principal axes, thereby altering the length scales in the cardinal directions. These length scales determine the possible positions and sizes of concentration peaks (whether single or multiple). To illustrate this, we repeated the simplified simulations discussed above, but on a static elliptical geometry (with the same area as the circular domain) and with the initial patch aligned along the shorter axis. The resulting montages are shown in Fig DA.

In Fig DA, we can observe how initiating the simulation on an elongated geometry alters the system dynamics. The most pronounced change occurs for the rotation pattern, which transforms into oscillation aligned with the long axis within a single rotation period ($< 200\,\mathrm{s}$). Initially, the patch travels clockwise, but upon reaching the curved end of the ellipse, its motion stalls. After one complete pass around the cortex, the characteristic rotation dynamics are no longer observed, having been replaced by oscillation. This transition occurs reproducibly when the axis ratio exceeds the threshold $a/b \sim 1.7$. Alignment with the long axis is evident in Fig DB, where the orange line settles around 0° first, earlier than for the other two patterns.

The remaining two patterns also align with the long axis, although on a longer time-scale. In Fig DB, we see that oscillation (purple line) undergoes two full periods ($\sim 500\,\mathrm{s}$) while gradually progressing toward alignment with the long axis, where it eventually settles. In the last row of Fig DA, we observe how this alignment affects static polarization: the patch slowly drifts toward one of the narrow ends of the ellipse, taking approximately $300\,\mathrm{s}$ of simulation time. This time-scale lies between the alignment times of the other two patterns, as shown in Fig DB. Once aligned, the patch remains fixed in that position.

3. Transport effects on RD dynamics

It has been well established [28] that diffusion plays a major role in determining which solutions are possible in an RD system. More precisely, it is the ratio of the time-scales of diffusion and reactions that governs the resulting patterns. To investigate the effects of transport in our RD model, we now examine how the solutions depend on the diffusion coefficients. For this purpose, we initiated two batches of simulations on a stationary circular geometry, using oscillation and rotation snapshots as the initial concentration profiles. The two initial conditions differ in the total protein content in the system: oscillation with $N_1 = 2300$ and $N_2 = 700$, and rotation with $N_1 = 4000$ and $N_2 = 1200$. The label 1 refers to combined number of molecules of activator "a", substrate "s" and complex "c", and the label 2 refers to combined molecule number of inhibitor "in" and complex "c".

To explore the effects of transport, we varied two surface diffusion coefficients: those of activator "a" and of the complex "c". Bulk diffusion coefficients are at least an order of magnitude higher than the surface values, making their variational effects negligible on the time-scales accessible to our simulations. To further support our choice of surface diffusions for this analysis, we recall the roles of these two protein species in our model. In RD models, it is common to compare the diffusion of the activator with that of the inhibitor or substrate. Accordingly, we selected the activator diffusion coefficient D_a as one of the variables. The complex "c", on the other hand, acts as a delayed inhibition mechanism: although it still promotes attachment of "a" from the cytoplasmic "s" pool, it does so at a rate an order of magnitude lower than "a" itself. Furthermore, it sequesters inhibitor "in" molecules, preventing them from participating in further reactions. The replenishment of cytoplasmic inhibitor "in" therefore depends directly on the transport of this complex around the cortex. Varying the two diffusion coefficients, D_a and D_c, allows us to subtly tune the overall dynamics of the RD system.

In Fig E, we present the limit-cycle patterns obtained by varying the two selected surface diffusion coefficients for the two initial concentration profiles described above. In both diagrams, the system exhibits a similar response. Increasing activator diffusion promotes the emergence of rotations. This arises because the oscillation pattern is highly sensitive to small asymmetries in the system, which scale with the transport rate. As introduced earlier, oscillation can be viewed as the superposition of two waves travelling in opposite directions around the cortex. If the system is perturbed (*e.g.*, by numerical noise), one of these waves typically overpowers the other. Furthermore, for very low activator diffusion ($D_\mathrm{a} = 0.01$, the smallest value on the diagram), we observe mesa-like concentration profiles.

The effect of increasing diffusion of complexes D_c is the stabilization of static polarization profiles at high values. In both diagrams, the limit cycle transitions to static polarization is at a threshold value of approximately 0.7.

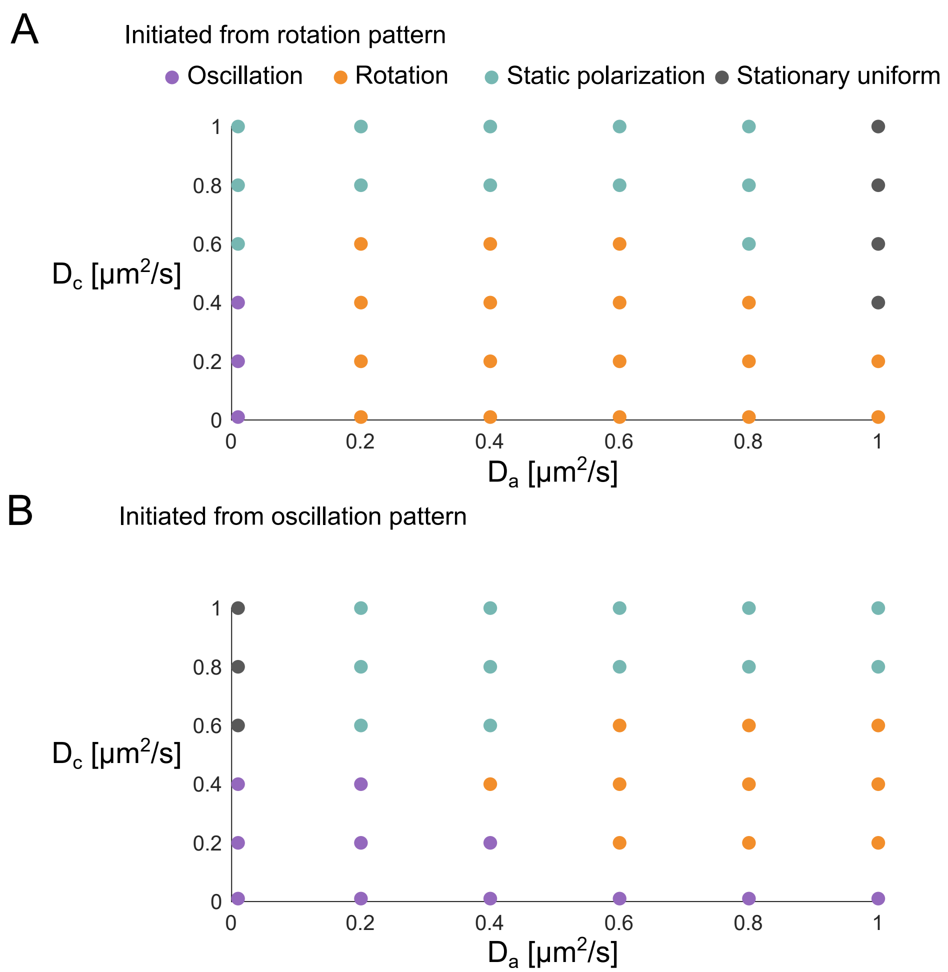

Fig E. Phase diagrams showing limit-cycle patterns on a static circular geometry for different values of surface diffusion coefficients. In both panels, activator cortex diffusion constant D_a is denoted on the horizontal axis, and complexes cortex diffusion constant D_c on the vertical axis. The smallest studied value for diffusion constants is 0.01. The **stationary uniform** state refers to a configuration in which all protein concentration profiles are uniform across their respective compartments and remain stable over time. Limit-cycle classification was determined after 1000 s of simulation time. (A) Phase diagram obtained from the rotation snapshot as the initial concentration profile, with $N_1 = 4000$ and $N_2 = 1200$. (B) Phase diagram obtained from the oscillation snapshot as the initial concentration profile, with $N_1 = 2300$ and $N_2 = 700$.

Furthermore, high activator and complex diffusion coefficients can push the system into a stationary uniform state, effectively shutting down the dynamics, when initiated with higher protein numbers and rotational concentration profiles. Conversely, the same effect occurs for low activator diffusion and high complex diffusion when initiated with lower protein numbers and oscillatory concentration profiles. In the former case, the over-abundance of activator molecules, coupled with their rapid diffusion, suppresses dynamic behaviour. In the latter case, the lack of protein molecules, combined with low transport of the activator, leads to the same outcome.

For the simulations presented in the main text, we used the values $D_\mathrm{a} = 0.3$ and $D_\mathrm{c} = 0.1$. This sets the system firmly apart from the phase transitions visible in Fig E.

[1] M. Patra and M. Karttunen, Stencils with isotropic discretization error for differential operators, Numer Methods Partial Differ Equations **22**, 936 (2006).
[2] Y. Oono and S. Puri, Computationally efficient modeling of ordering of quenched phases, Phys Rev Lett **58**, 836 (1987).
[3] Y. Oono and S. Puri, Study of phase-separation dynamics by use of cell dynamical systems. i. modeling, Phys Rev A **38**, 434 (1988).
[4] F. H. Harlow and J. E. Welch, Numerical calculation of time-dependent viscous incompressible flow of fluid with free

surface, Phys Fluids **8**, 2182 (1965).
[5] A. Chorin, Numerical solution of the navier–stokes equations, Math Comput **22**, 745 (1968).
[6] S. Parameswaran and J. C. Mandal, A stable interface-preserving reinitialization equation for conservative level set method, Eur J Mech B Fluids **98**, 40 (2023).
[7] The MathWorks, Inc., *MATLAB (R2023b)*, The MathWorks, Inc., Natick, Massachusetts (2023), [Computer software].
[8] J. Schindelin, I. Arganda-Carreras, E. Frise, V. Kaynig, M. Longair, T. Pietzsch, S. Preibisch, C. Rueden, S. Saalfeld, B. Schmid, J.-Y. Tinevez, D. J. White, V. Hartenstein, K. Eliceiri, P. Tomancak, and A. Cardona, Fiji: An open-source platform for biological-image analysis, Nat Methods **9**, 676 (2012), fiji/ImageJ v1.54p was used in this study.
[9] P. Baniukiewicz, S. Collier, and T. Bretschneider, Quimp: Analyzing transmembrane signalling in highly deformable cells, Bioinformatics **34**, 2695 (2018), quimP module for Fiji/ImageJ (accessed 23 May 2025).
[10] G. A. Dunn and A. F. Brown, Quantifying cellular shape using moment invariants, in *Biological Motion*, Lecture Notes in Biomathematics, Vol. 89, edited by W. Alt and G. Hoffmann (Springer, Berlin, Heidelberg, 1990) pp. 10–34.
[11] M. R. Teague, Image analysis via the general theory of moments, J Opt Soc Am **70**, 920 (1980).
[12] W. Feneberg, M. Westphal, and E. Sackmann, Dictyostelium cells' cytoplasm as an active viscoplastic body, Eur Biophys J **30**, 284 (2001).
[13] L. T. S. Nguyen and D. N. Robinson, The lectin discoidin i acts in the cytoplasm to help assemble the contractile machinery, J Cell Biol **221**, e202202063 (2022).
[14] Y. Hayashida, Y. Gomibuchi, C. Oosawa, T. Yasunaga, and Y. V. Morimoto, Establishing functional giant Dictyostelium cells reveals front–rear polarity in intracellular signaling, Commun Biol **9**, 71 (2026).
[15] E. O. Potma, W. P. de Boeij, L. Bosgraaf, J. Roelofs, P. J. M. van Haastert, and D. A. Wiersma, Reduced protein diffusion rate by cytoskeleton in vegetative and polarized dictyostelium cells, Biophys J **81**, 2010 (2001).
[16] A. Abu-Arish, P. Kalab, J. Ng-Kamstra, K. Weis, and C. Fradin, Spatial distribution and mobility of the Ran GTPase in live interphase cells, Biophys J **97**, 2164 (2009).
[17] B. D. Slaughter, J. R. Unruh, A. Das, S. E. Smith, B. Rubinstein, and R. Li, Non-uniform membrane diffusion enables steady-state cell polarization via vesicular trafficking, Nat Commun **4**, 1380 (2013).
[18] B. Khalili, L. Merlini, V. Vincenzetti, S. G. Martin, and D. Vavylonis, Exploration and stabilization of ras1 mating zone: A mechanism with positive and negative feedbacks, PLOS Comput Biol **14**, e1006317 (2018).
[19] S. Das, T. Yin, Q. Yang, J. Zhang, Y. I. Wu, and J. Yu, Single-molecule tracking of small gtpase rac1 uncovers spatial regulation of membrane translocation and mechanism for polarized signaling, PNAS **112**, E267 (2015).
[20] P. H. M. Lommerse, B. E. Snaar-Jagalska, H. P. Spaink, and T. Schmidt, Single-molecule diffusion measurements of h-ras at the plasma membrane of live cells reveal microdomain localization upon activation, J Cell Sci **118**, 1799 (2005).
[21] M. Elsner, H. Hashimoto, J. C. Simpson, D. Cassel, T. Nilsson, and M. Weiss, Spatiotemporal dynamics of the COPI vesicle machinery, EMBO Rep **4**, 1000 (2003).
[22] D. Brough, F. Bhatti, and R. F. Irvine, Mobility of proteins associated with the plasma membrane by interaction with inositol lipids, J Cell Sci **118**, 3019 (2005).
[23] M. Šoštar, *Analiza dinamike proteina Rac1 tijekom staničnog kretanja*, Phd thesis, Sveučilište u Zagrebu, Prirodoslovno-matematički fakultet, Zagreb, Hrvatska (2022), disertacija, otvoreni pristup.
[24] B. Ivšić, *Dinamika proteina Rac1 te njena ovisnost o obliku stanice*, Master's thesis, Faculty of Science (PMF), University of Zagreb, Department of Physics, Zagreb, Croatia (2019), in Croatian.
[25] M. Šoštar, M. Marinović, V. Filić, N. Pavin, and I. Weber, Oscillatory dynamics of rac1 activity in *Dictyostelium discoideum* amoebae, PLOS Comput Biol **20**, e1012025 (2024).
[26] J. Halatek, F. Brauns, and E. Frey, Self-organization principles of intracellular pattern formation, Philos Trans R Soc London, Ser B **373**, 20170107 (2018).
[27] K. S. Eroumé, A. Vasilevich, S. Vermeulen, J. de Boer, and A. Carlier, On the influence of cell shape on dynamic reaction–diffusion polarization patterns, PLOS One **16**, e0248293 (2021).
[28] A. M. Turing, The chemical basis of morphogenesis, Philos Trans R Soc London, Ser B **237**, 37 (1952).
[29] Y. Mori, A. Jilkine, and L. Edelstein-Keshet, Wave-pinning and cell polarity from a bistable reaction–diffusion system, Biophys J **94**, 3684 (2008).
[30] A. Gierer and H. Meinhardt, A theory of biological pattern formation, Kybernetik **12**, 30 (1972).
[31] H. Meinhardt and A. Gierer, Generation and regeneration of sequence of structures during morphogenesis, J Theor Biol **85**, 429 (1980).
[32] A. R. Singh, T. Leadbetter, and B. A. Camley, Sensing the shape of a cell with reaction diffusion and energy minimization, PNAS **119**, e2121302119 (2022).
[33] R. Geßele, J. Halatek, L. Würthner, and E. Frey, Geometric cues stabilise long-axis polarisation of PAR protein patterns in *C. elegans*, Nat Commun **11**, 539 (2020).
